# Discourse accessibility constraints in children’s processing of object relative clauses

**DOI:** 10.3389/fpsyg.2015.00860

**Published:** 2015-06-23

**Authors:** Yair Haendler, Reinhold Kliegl, Flavia Adani

**Affiliations:** ^1^Department of Linguistics, University of Potsdam, PotsdamGermany; ^2^Department of Psychology, University of Potsdam, PotsdamGermany

**Keywords:** child language, relative clauses, discourse, pronouns, intervention locality, visual-world paradigm

## Abstract

Children’s poor performance on object relative clauses has been explained in terms of intervention locality. This approach predicts that object relatives with a full DP head and an embedded pronominal subject are easier than object relatives in which both the head noun and the embedded subject are full DPs. This prediction is shared by other accounts formulated to explain processing mechanisms. We conducted a visual-world study designed to test the off-line comprehension and on-line processing of object relatives in German-speaking 5-year-olds. Children were tested on three types of object relatives, all having a full DP head noun and differing with respect to the type of nominal phrase that appeared in the embedded subject position: another full DP, a 1st- or a 3rd-person pronoun. Grammatical skills and memory capacity were also assessed in order to see whether and how they affect children’s performance. Most accurately processed were object relatives with 1st-person pronoun, independently of children’s language and memory skills. Performance on object relatives with two full DPs was overall more accurate than on object relatives with 3rd-person pronoun. In the former condition, children with stronger grammatical skills accurately processed the structure and their memory abilities determined how fast they were; in the latter condition, children only processed accurately the structure if they were strong both in their grammatical skills and in their memory capacity. The results are discussed in the light of accounts that predict different pronoun effects like the ones we find, which depend on the referential properties of the pronouns. We then discuss which role language and memory abilities might have in processing object relatives with various embedded nominal phrases.

## Introduction

### Relative Clause Processing in Children and Adults

The acquisition of relative clauses has been studied extensively and in a large variety of languages (Brandt et al., 2009; Arnon, 2010; Adani, 2011; Arosio et al., 2012; Belletti et al., 2012; Adani et al., 2014, among others). The existing research focuses mainly on the asymmetry between child performance on subject-extracted relatives (SRs) and object-extracted relatives (ORs), examples of which are provided in (1) and (2), respectively. In the examples, the head of the relative clause is the noun it modifies (*the bunny*). The underscore marks the position in the embedded clause from which the head noun is extracted: subject position in SRs and object position in ORs.

(1) The bunny that __ is chasing the horse
(2) The bunny that the horse is chasing __

In head-initial languages, it is a robustly attested finding that young children have difficulties comprehending and producing ORs, but not SRs (see Gutierrez-Mangado, 2011 for a reversed pattern in Basque). Children’s errors with ORs are mainly expressed by the interpretation of these sentences as SRs. An account that aims to explain the SR–OR asymmetry in acquisition is proposed by Friedmann et al. (2009), following earlier work by Grillo (2005, 2009). This approach provides an explanation in terms of intervention locality, based on the syntactic principle of Relativized Minimality (RM; Rizzi, 1990 and subsequent work). We will refer to Friedmann et al.’s (2009) approach as the RM account.

Relativized Minimality is based on the configuration in (3), in which X is a constituent that moves from its original (gap) position Y crossing an intervening constituent Z.

(3) X … Z … Y

According to the RM Principle, a local relation between X and Y is impossible if Z is a potential candidate for that local relation. Such a case occurs when Z intervenes between X and Y and when Z is structurally similar to X. These two co-occurring conditions give rise to a locality intervention effect and, thus, to difficulties in parsing the structure. Friedmann et al. (2009) show how this configuration and the conditions that create intervention effects apply to the structure of SRs and ORs ^1^. In the case of relative clauses, the authors identify the feature [+NP], or ‘lexical restriction,’ as the one that, when present on both X and Z, makes them structurally similar. In (1) and (2), repeated as (4) and (5), both X and Z are lexically restricted, or in other words: they are both full DPs. But only in the OR Z intervenes between X and Y. For this reason, according to Friedmann et al. (2009) ORs with two full DPs are difficult for children whereas SRs with two full DPs are not.


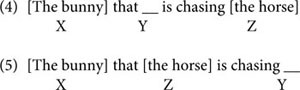


The RM account predicts significant improvement in child comprehension of ORs when the head (X) is a full DP, whereas the embedded subject (Z) is not. Children are therefore predicted to perform more accurately on an OR with a full DP head and an embedded subject which is a personal pronoun, a DP that lacks the [+NP] feature. Friedmann et al. (2009, p. 75) tested this prediction examining child comprehension of Hebrew ORs with an embedded subject which is a null pronoun. The following example is taken from their paper.

(6) Tare li et ha-sus she- mesarkim oto show to-me ACC the-horse that- *pro* brush-3rd-pl him ‘Show me the horse that someone is brushing’ (literally, ‘the horse that they are brushing’)

The Hebrew *pro* subject in (6) is an impersonal subject that agrees with the 3rd-person plural form, as evidenced by the Person and Number agreement marking on the embedded verb *brush*. This impersonal, or arbitrary *pro* is used to describe the action of an unspecified agent. Friedmann et al. (2009) found that children understood ORs like (6) more accurately than ORs with a full DP head noun and a full DP embedded subject. They explained the improved comprehension as due to the attenuation of the intervention locality effect, caused by the fact that the head of the OR is a full DP but not its embedded pronominal subject. Crucially, the prediction is that any type of pronoun in the embedded subject position will improve comprehension, since what matters is the lack of lexical restriction, a property shared by all personal pronouns. This prediction receives further support from studies that find relatively accurate child performance on ORs whose embedded subject is an overt 3rd-person pronoun (Brandt et al., 2009), a 2nd-person pronoun (Kidd et al., 2007) or a 1st-person pronoun (Arnon, 2010).

Other accounts that explain OR processing based on adult performance make similar predictions. Warren and Gibson (2002, 2005) propose that sentence processing is determined by the number of new referents that intervene between a moved element (filler) and the gap site in which it is integrated into the structure. The greater the number of intervening referents (e.g., noun phrases, verbs) the harder it is to keep track of the filler until the gap site is encountered and the filler-gap dependency is resolved [a similar idea is advanced by O’Grady (2011)]. Under this view, an intervening pronoun reduces processing cost since it does not introduce a new discourse referent: it serves as a link to an already given one. Indeed, adults have less difficulty with doubly nested ORs and object clefts whose embedded-most DP is a pronoun, as compared to cases in which all the nominal phrases in the structure are full DPs (Warren and Gibson, 2002, 2005). Other accounts explain the difficulty with ORs in terms of similarity between the DP head and the embedded subject DP. It has been found that an OR becomes easier to parse when these two constituents are sufficiently dissimilar. For instance, ORs with two full DPs are more costly to process than ORs in which the head is a full DP and the embedded subject is a proper name (Gordon et al., 2004), or a 2nd-person pronoun (Gordon et al., 2001). Other studies define the difficulties with OR processing in terms of cue-based interference (Lewis and Vasishth, 2005; Lewis et al., 2006; van Dyke and McElree, 2006). Under this view, the similarity between the DP head and the embedded DP is defined by the cues that these two constituents bear. When a constituent (e.g., the DP head in an OR) is encountered it is encoded in memory. Later on, in the gap position, it has to be retrieved from memory in order to be integrated into the structure. At this point, its (syntactic, semantic, or other) cues are analyzed in order to decide whether the filler-gap dependency can be resolved. If another constituent (e.g., the embedded subject DP in an OR) shares similar cues with those of the encoded constituent this second set of cues will interfere with the processing of the first one, increasing the overall processing cost of the structure. In an OR with an embedded pronoun, the cues of the intervening pronoun are sufficiently different from those of the encoded head noun, thus reducing the processing cost.

As can be seen, there is an affinity between the RM account and the accounts reviewed in the last paragraph, although the former is the only one whose predictions have been tested in experiments with children. All these accounts appear to share the prediction that an OR with an embedded pronominal subject is less costly for processing than an OR in which both the head noun and the embedded subject are full DPs. Moreover, at least some of these approaches (Gordon et al., 2001; Lewis et al., 2006), like the RM account, attribute the difficulties in OR processing to the (dis)similarity between the DP head and the embedded subject DP in terms of cues or features. Importantly, however, each of these studies tested the effect of only one pronoun type on OR processing. The only exception is Warren and Gibson’s (2002) study with adults, to which we will return later. The present study is the first to assess the comprehension of ORs with different embedded pronominal subjects in children. That is, we will test the prediction that ORs with different pronouns in the embedded subject position should be equally easy for children, as compared to ORs with two full DPs. Comparing the effects of different pronoun types is particularly interesting given studies that show that pronouns with different referential properties affect sentence processing differently in adults (Warren and Gibson, 2002; Carminati, 2005).

We have recently shown (Haendler et al., 2015) that there is a relation between children’s performance on ORs with different types of embedded referring expressions (full DP, different personal pronouns) ^2^ and their language skills, as measured by standardized tests for receptive grammatical abilities. These language or grammatical skills (we will use the two terms interchangeably) were defined as the average score on three subtests from Siegmüller et al. (2010). The tests assessed the comprehension of (a) canonical and non-canonical declarative sentences (SVO and OVS); (b) sentences containing reflexives and pronouns; (c) various types of relative clauses (right-branching and center-embedded; SRs and ORs). In the discussion, we will elaborate on what grammatical skills are assumed to underlie children’s performance on these three language tests. Concerning the results, we found that children were most accurate on ORs with an embedded 1st-person pronoun (OR + 1pro; *The horse that I chase*), independently of their scores on the language tests. In ORs with an embedded 3rd-person pronoun (OR + 3pro; *The horse that it chases*) and ORs with a full DP head and an embedded full DP (OR + 2DP; *The horse that the bunny chases*), which were overall more difficult, children’s performance interacted with their grammatical skills: children with higher scores on the language tests were more accurate on these conditions than children with lower scores.

In the present paper, we extend this picture by looking at memory skills and assessing whether they interact with language abilities in the modulation of children’s performance on the three OR types. In other words, we want to see whether both language and memory have an impact on children’s OR processing, and whether their effects are independent of one another or whether they interact. In the latter case, we want to see what kind of relation between language and memory skills emerges during OR processing. This kind of analysis will help distinguish between effects that are purely due to children’s language skills, effects that are purely memory-dependent and effects that are caused by both types of cognitive abilities.

### Memory and the Processing of Object Relative Clauses

The relevance of memory for the processing of relative clauses has been vastly investigated. To begin with, Friedmann et al. (2009) speculate that the difficulty with an OR containing two full DPs lies in children’s limited memory capacity. During the processing of such a structure, one needs to hold in memory the featural specifications of the DP head and the embedded DP and compare them in order to determine their (dis)similarity (see also Adani et al., 2010). When the features of the DP head and of the embedded DP are similar, such as when they are both full DPs, the comparison of the features is more costly and memory capacity is overloaded. However, when the features on the DP head and on the embedded DP are sufficiently different, as in the case of an OR with an embedded pronominal subject, comparing the features becomes less demanding for memory resources and the comprehension of the OR is facilitated.

The reviewed accounts on adult processing similarly suggest that memory abilities constrain the processing of ORs (for a comprehensive review, see Wagers and Phillips, 2014). According to Gibson (1998, 2000) and Warren and Gibson (2002, 2005; see also O’Grady, 2011), the difficulty associated with keeping track of the filler while processing newly introduced discourse referents is related to available memory resources. The greater the number of new discourse referents that intervene between the filler and its gap site, the longer the filler has to be kept in memory until the filler-gap dependency is resolved. Therefore, people with strong memory capacity will be facilitated in maintaining the filler in memory while processing the sentence until the gap position is reached. Gordon et al.’s (2001, 2004) proposal that the processing cost of an OR is determined by the (dis)similarity between the DP head and the embedded DP is also related to memory capacity. The idea is that dissimilar DPs burden memory to a lesser extent, making the distinction of the two constituents during sentence processing easier. Finally, the processing mechanism assumed under the cue-based interference account (Lewis and Vasishth, 2005; Lewis et al., 2006; van Dyke and McElree, 2006) similarly draws on memory resources. If the set of cues of a previously encoded constituent (the DP head of an OR) and that of the intervening DP are similar, memory capacity will be overloaded, resulting in an increased processing cost. If the two sets of cues are dissimilar, memory resources will be less burdened and the sentence will be easier to process.

The relation between children’s memory abilities and their comprehension of syntactically complex sentences has been vastly studied. Different studies have used different kinds of tests to measure memory, yielding mixed results. Some studies found a relation between children’s off-line response accuracy and their performance on listening span tasks (Montgomery et al., 2008; Montgomery and Evans, 2009; Weighall and Altmann, 2011), backward digit span tasks (Engel de Abreu et al., 2011; Boyle et al., 2013) and forward digit span tasks (Arosio et al., 2011, 2012; Engel de Abreu et al., 2011). An association has been found also between similar memory tasks and children’s on-line sentence processing (Booth et al., 2000; Roberts et al., 2007). However, no systematic relation has been found between the score on any specific memory test and children’s performance on any specific language task (Kidd, 2013). Particularly relevant for the present study is Arosio et al.’s (2012) work. Using a picture-selection task, they tested 7-years-old German-speaking children on the comprehension of SRs and ORs, disambiguated either by case-marking on the determiner of the embedded DP or by number-marking on the embedded verb. The authors found that children were more accurate on case-disambiguated than on number-disambiguated ORs. Also relevant is their finding that children’s score on a forward span test was a reliable predictor of their comprehension of ORs (but not SRs).

In the present study, we administered to children both a forward and a backward digit span task. The memory measure was calculated as the average score on the two tests. As we have seen, both the forward and the backward span tests have been widely used in studies with children. Moreover, these tasks are typically assumed to reflect two kinds of memory components in Baddeley’s classical model (Baddeley, 1986; Baddeley et al., 2009): the forward digit span task is believed to reflect the operation of the phonological loop, a short-term storage of phonological information; the backward digit span task is assumed to reflect the operation of the central executive, which is responsible for the coordination and elaboration of the stored information. The former is often referred to as *verbal short-term memory*; the latter as *verbal working memory* (Kidd, 2013). The fact that no systematic relation has been demonstrated between any of these two tests and a specific performance pattern on language comprehension led us to combine the scores on the two tasks into one, more general measure of memory capacity. The disadvantage in doing so is that we cannot look at separate effects caused by the two kinds of memory abilities (short-term memory and working memory). The advantage is that such a general memory measure is more robust and reliable for the analysis, since it combines data collected in two different tasks. The mixed findings in the literature regarding the relation between the two span tasks and certain language abilities leaves the qualitative analysis of the role of memory highly speculative. Hence, by using the composite score, we gain a stronger measure for the quantitative analysis of children’s memory capacity.

### Referential Properties and Discourse Accessibility

As we have seen, the prediction we are testing is that any type of embedded pronoun should facilitate children’s performance on ORs to an equal extent. However, there is extensive literature focusing on differences between pronouns in terms of their referential properties. A case in point is the different way of establishing reference of 1st- and 2nd-person pronouns on the one hand, and 3rd-person pronouns on the other hand. When a participant in a linguistic act constructs a discourse model, 1st- and 2nd-person pronouns are directly integrated into that model since they refer, respectively, to the speaker and the interlocutor, two discourse referents which are always available and highly accessible (Recanati, 1993; Erteschik-Shir, 1997; Ariel, 2001). Moreover, the referents of these pronouns are derived from the lexical meaning of the pronouns themselves: 1st-person pronoun (‘I,’ ‘we’) = speaker; 2nd-person pronoun (‘you’) = interlocutor. This is similar to the way in which a regular noun phrase (e.g., ‘the horse’) establishes reference. The discourse referent of the noun phrase is derived from its lexical meaning, despite the fact that it is marked with 3rd-person (unlike 1st- and 2nd-person pronouns) and although it is not referring to a participant in the linguistic act (like ‘speaker’ or ‘interlocutor’). By contrast, the referent of a 3rd-person pronoun (‘it,’ ‘they,’ and demonstratives such as ‘this,’ ‘that’) is derived from the discourse, in a process of pronoun resolution in which the pronoun relates to an antecedent in the linguistic or extra-linguistic context (Heim, 1991; Legendre and Smolensky, 2012).

There is experimental evidence that such differences in discourse accessibility of pronouns affect the processing of sentences in which they occur. Warren and Gibson (2002) found that adults perceive doubly nested ORs with an embedded 1st- or 2nd-person pronoun as less complex, as compared to such structures with an embedded 3rd-person pronoun. Moreover, adult on-line processing of pronoun resolution in infrequent circumstances (when the pronoun antecedent is a previously mentioned object, rather than subject) is facilitated when that pronoun is marked with 1st- or 2nd-person, rather than 3rd-person (Carminati, 2005). These effects, assumed to be caused by the referential properties of pronouns, have not been tested yet in children. But a number of studies suggest children are sensitive to discourse properties of pronouns as well. First, in line with the pronoun asymmetry described above, children acquire the ability to correctly interpret 1st- and 2nd-person pronouns before 3rd-person pronouns (Brener, 1983; Girouard et al., 1997; Legendre et al., 2011; Legendre and Smolensky, 2012). Moreover, there is substantial evidence indicating that children are sensitive to the discourse properties that determine pronoun usage and interpretation (Song and Fisher, 2005, 2007; Spenader et al., 2009; Pyykkönen et al., 2010; Koster et al., 2011; Hartshorne et al., 2015) ^3^. For instance, Song and Fisher (2005) found that 3-year-olds, tested with a preferential-looking paradigm, looked more to the correct referent figure of a pronoun when it was made prominent in the discourse (in the preceding context it was the first-mentioned figure in a subject position and pronominalized once), than when the referent was not prominent. Children in Koster et al.’s (2011) study interpreted the pronoun as referring to the first-mentioned character in a context story, both when this character was consistently the discourse topic and when there was a shift in the topic of the story. Production studies also suggest that children are sensitive to referential properties of pronouns, as well as to the extra-sentential or extra-linguistic context, when they choose which referring expression to utter (see Serratrice, 2013 and references therein). Together, these studies suggest that, from early on, children are sensitive to discourse properties of pronouns such as topicality or order-of-mention. It appears that children can use these properties in order to construct a plausible discourse model and, based on that model, derive expectations regarding the usage of the referring expressions they encounter in the linguistic input (see a related discussion in Trueswell et al., 2011).

According to Goodluck (2010), who discusses data in contradiction with Friedmann et al.’s (2009) approach, children’s performance on complex structures is determined by both syntactic and discourse accessibility operations (see also Goodluck, 1990, 2005 and Avrutin, 2000). Whereas the RM account predicts difficulties with object-extracted wh-questions in which both the moved constituent and the intervening one are full DPs (*Which lion did the zebra kick?*), Goodluck (2005) found that children perform more accurately when the moved constituent is a more generic name (*Which animal did the zebra kick?*). In explaining the data, Goodluck suggests that children’s difficulty with object *which*-questions is related both to the syntactic factor of distance (*which lion/animal* is extracted from the more distant position as the object of the verb *kick*) and to the discourse factor of set-restriction (to interpret *which lion*, the child has to restrict the set of given lions and understand which one she is asked about; this operation is less costly when *lion* is replaced with the more generic *animal*). Although Goodluck’s (2010, p. 1520) proposal is made in relation to structures that are slightly different from the ones dealt with here, the relevance of her work lies in the idea that “[…] children appear to have difficulty in general with grammatical phenomena that require access to discourse.”

### The Present Study

To summarize the goal of the present study, we test the prediction that ORs with different embedded pronominal subjects are easier than ORs with two full DPs. Moreover, no difference is predicted between the conditions with pronouns. We used right-branching ORs with various referring expressions in the embedded subject position. ORs with an embedded 1st-person pronoun (7) and with 3rd-person pronoun (8) were compared to a baseline condition of ORs in which both the head noun and the embedded subject are full DPs (9) ^4^. Note that these ORs differ with respect to the referring expression that occupies the embedded subject position (in bold). Hence, we expect differences in performance on the ORs to reflect effects caused by these referring expressions.


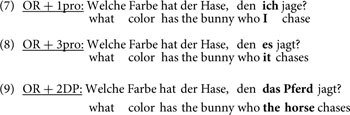


Previous studies on children’s OR comprehension have used only off-line methods. Here, we designed a visual-world experiment (Tanenhaus et al., 1995) and measured both off-line response accuracy and on-line eye-gaze during the inspection of a visual scene that accompanied each test sentence. The off-line accuracy was collected as a measure of explicit comprehension; the on-line eye-gaze as a measure of implicit parsing strategies. Many studies using on-line measures (e.g., eye-tracking) have found evidence for early processing of complex structures and/or a more fine-grained performance pattern that usually remains hidden in the explicit response (Brandt-Kobele and Höhle, 2010; Adani and Fritzsche, 2015). Thus, on-line gaze measures are arguably more sensitive in testing child language, yielding results that suggest that children might implicity process a structure accurately even when their explicit response is inaccurate. For this reason, and since previous studies have found difficulties with ORs that persist until late in development (e.g., Friedmann et al., 2009; Arosio et al., 2012; Adani et al., 2014), we tested children at age 5. If the on-line eye-gaze measure is indeed more sensitive than the off-line response accuracy we might find evidence for correct processing of the harder condition(s) even as early as this age.

Let us now summarize the predictions regarding children’s performance on the three conditions and the possible relation to language and memory abilities. The initial prediction is that children will be more accurate on OR + 1pro and OR + 3pro than on OR + 2DP, and there should be no difference between performance on OR + 1pro and OR + 3pro. However, if the different ways with which the 1st- and the 3rd-person pronouns establish reference influence children’s performance, as found with adults (Warren and Gibson, 2002; Carminati, 2005), children should be more accurate on OR + 1pro than on OR + 3pro. We have already mentioned that stronger grammatical skills improve children’s performance on two of the conditions. Given previous studies (Kidd, 2013), we might expect to find also an impact of memory that shows that stronger memory capacity improves performance on the task. We might also find that language and memory abilities modulate children’s performance differently. This would result in different patterns of interaction between language/memory and response accuracy/eye-gaze.

Regarding the specific pattern expected in the two kinds of data we have collected, a higher proportion of correct responses (i.e., naming the color of the correct figure) will express a more accurate off-line performance. With respect to the eye-gaze data, there are several possibilities. We measure the proportion of looks to the target figure in the visual scene that accompanies each test sentence, within a time window defined in advance for the analysis. Accurate processing of the sentence within the analysis window will be expressed either by earlier looks to the target figure, or by longer looks to the target (higher proportion of target looks), or both. Therefore, the initial predictions regarding the performance pattern in the accuracy data and the eye-gaze data roughly correspond. However, we might find evidence for correct processing of the sentences, or a more fine-grained performance pattern, only in the eye-gaze data.

## Materials and Methods

### Participants

Forty-seven 5-years-old children (24 females, age range 5.0–5.11, *M* = 5.5) participated in the study. All children are growing up as monolingual speakers of German and none has reported history of linguistic, hearing or other cognitive developmental disorders. Parents gave their consent for the participation of their children. The study, approved by the ethics commission of the University of Potsdam, was successfully piloted with a group of university students.

### Material

#### Visual Stimuli

**FIGURE 1:**
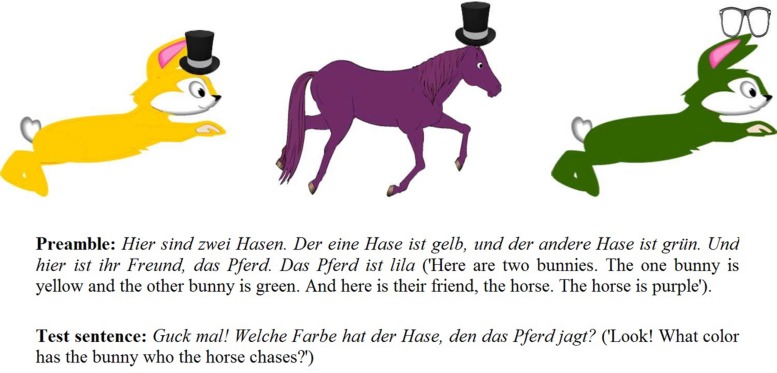
**Example of a visual scene, a preamble and a test sentence**.

In a setup inspired by Arnon (2005, 2010) and Adani (2011) participants watched in each trial an animated video with two identical animals on the sides (target and distractor animals) and a third different animal in the middle (middle animal). Each of these three regions of interest had the same size of 436 × 400 pixels. An example of a visual scene is provided in **Figure 1**. Employing two verbs, ‘chase’ and ‘tickle,’ the three animals in the scene were chasing each other on half of the trials and tickling one another with a feather on the other half. Each of the animals in the scene was colored differently. The three colors were combined such that similar colors did not appear within the same video, in order to facilitate color distinction and recognition (Pitchford and Mullen, 2003). Each of the animals carried a small object (hat, glasses, flower or heart–all clip art images) that was relevant for the fillers, but not for the experimental items. The target animal (i.e., the referent of the OR head noun) could be one of four masculine nouns–bear, bunny, lion, or monkey–each of which appeared an equal number of times as target, and in a balanced manner across conditions. The middle animal was on some trials a neuter noun (horse, camel, zebra, or sheep) and on others a feminine noun (duck, cow, cat, or mouse). In the OR + 1pro condition, the middle animal was always the dog, established as referent for the 1st-person pronoun in an introduction story prior to the experiment (see Procedure). The direction of the scene was in half of the trials from left to right and in the other half from right to left. Depending on the action direction, the target animal was always either on the left or on the right side of the scene, but never in the middle. In the ORs, the target animal was always the last animal in the row; in the fillers, it was always the first animal in the row, to prevent participants from anticipating the side on which the target appeared.

#### Linguistic Stimuli

The design consisted of three experimental conditions [examples (7)–(9) in the Introduction], with seven trials in each condition, and 12 fillers (e.g., *Welche Farbe hat der Hase mit dem Hut?* ‘What color is the bunny with the hat?’). Piloting the experiment before the actual testing revealed that, with this amount of items, the duration of the experiment (∼20 min) was adequate for 5-year-olds. The displayed videos were accompanied by the test sentences that were pre-recorded with a female German native speaker and integrated into the video file. These were questions about the color of one animal in the scene to be identified through a relative clause (in experimental items) or a small object (in fillers). Two lists were constructed, each containing a different pseudo-randomized order of the items. Half of the participants were exposed to the first list, and the other half were exposed to the second list. The full list of items is provided in the online supplementary material.

Since all the target animals (i.e., the OR head noun) were singular masculine nouns, the relative pronoun in all the ORs was always unambiguously accusative case-marked (*den*, ‘who__ACC___MASC_’). This way, the sentence is revealed to be an OR already upon encountering the relative pronoun and children might be facilitated in processing the sentence (Arosio et al., 2012). However, in order for children to be able to make use of this information, they have to be able to recognize the accusative case-marking on the relative pronoun. In particular, they have to be able to distinguish the accusative case-marked *den* from the nominative case-marked *der*. If children cannot tell apart the two minimally differing case-markings they might erroneously understand the sentence as a SR (e.g., *Welche Farbe hat der Hase, der das Pferd jagt?* ‘What color has the bunny who__NOM___MASC_ the horse chases?’). This might mask the comprehension difficulties children typically have with the syntactic structure of the OR as such. In order to determine whether children were able to discern between the two case-markings, we looked at their performance on one of the language tests that were administered (from the TSVK battery, Siegmüller et al., 2010): the test on the comprehension of OVS sentences, which are grammatical but non-canonical in German. Successful performance on this test requires the distinction between nominative (*der*), accusative (*den*) and dative case-marking (*dem*), in order to understand that the pre-verbal noun is an accusative- or dative-marked object and that the post-verbal noun is a nominative-marked subject. When looking at the performance on this test it appears that 37 out of 41 children scored at or above 50% (answering correctly six or more out of the 12 questions in the test). Scatterplots showing the relation between individual performance on this test and the overall performance in the experiment (both in terms of off-line accuracy and on-line eye-gaze) are provided in the online supplementary material. Additional evidence that children in our study were able to tell apart nominative and accusative case-marking stems from independent studies that show that children as old as 4.6 can already distinguish nominative and accusative case-marking in German (Grünloh et al., 2011) ^5^.

#### Memory

We administered to the children a forward span test and a backward version of the same test. The sequences for the forward span test were taken from the Intelligence and Development Scales battery (Grob et al., 2009). The forward span test was used to measure verbal short-term memory. To measure verbal working memory, we used the same sequences in a backward span test which is typically taken to measure this type of memory capacity. The sequences in the two memory tasks were of increasing length, ranging from 2 to 7 items in each sequence, and containing either digits or letters (for instance, 5-3-8 or C-O-G). For each sequence length (of two items, three items, and so on) there was one sequence of digits and one sequence of letters.

#### Language

The language tests were three subtests from Siegmüller et al.’s (2010) standardized battery for receptive grammatical abilities in German: subtest 3 for the comprehension of SVO and OVS sentences (e.g., *Die kinder zeichnet der Mann* ‘The__ACC_ children draws the__NOM_ man’); subtest 5 for the comprehension of sentences containing reflexives and pronouns (*Der Papa wäscht ihn* ‘The__NOM_ father washes him__ACC_’); and subtest 6 for the comprehension of various types of relative clauses (right-branching SR: *Den Hasen schiebt der Esel, der weint* ‘The__ACC_ bunny pushes the__NOM_ donkey that__NOM_ cries’; center-embedded OR: *Der Mann, den der Indianer trägt, liest* ‘The__NOM_ man, that__ACC_ the__NOM_ Indian carries, reads’). In all these tests, the task is to point to one picture out of three that best corresponds to a sentence read aloud by the experimenter.

### Procedure

The experiment was carried out at a university lab, in a quiet and child-friendly room. Participants were seated at a distance of 55–70 cm from a DELL laptop (screen resolution 1600 × 900, white background), connected to an SMI RED-m eye-tracker (sample rate 60 Hz). The experiment was run over the SMI Experiment Center software. An experimenter sat next to the participant, observing the tracking quality on a separate monitor and moving from one trial to the next, or repeating a trial if necessary, by pressing keys on an external keyboard. The experimenter also registered by hand the participant’s verbal response in each trial.

In an introduction video, displayed prior to the experiment, Nellie the dog appeared and explained she would like to have the child’s help in learning the color names. She explained the task and gave three example questions that served as warm-up trials. Participants received feedback on their responses to the practice trials, but not during the actual experiment. After the warm-up items, Nellie showed and named all the animals as well as the actions (chasing and tickling) that would appear in the game. The story teller also said she would appear every now and then and play with her friends. This, together with the appearance of the dog as the middle animal in the relevant trials, established the referent for the 1st-person pronoun and made its usage felicitous.

In the experiment, each trial started with a preamble video in which the animals of the scene were presented and their colors were named. The referent of the 3rd-person pronoun was stressed prosodically in the preamble, in order to make it more salient in the discourse. The test question followed the preamble video immediately (**Figure 1** shows an example of a visual scene with the preamble text and the test sentence accompanying it. An example of a preamble text and a test sentence for each of the conditions, as well as a video exemplifying a trial, can be found in the online supplementary material.). Upon hearing the question about the color of one of the animals, participants answered and the experimenter noted their response on a sheet. In case of no response the experimenter offered the participant to listen again to the question. In such cases, both the preamble and the test question were replayed and only the second response was counted in the analysis. A short break was taken after every 10 items. The entire duration of the experiment was approximately 20 min. Children, who were generally engaged and happy to participate, received stickers as a reward.

The forward and backward span tasks and the language tests were administered in a separate session, 1–3 weeks after the first appointment, at the same room at the university lab. The instructions for the forward span task were given following the protocol of this test (IDS, Grob et al., 2009). The instructions for the backward span task were based on those given in another such test that has norms from older children (HAWIK, Petermann and Petermann, 2008). In the forward span task, the experimenter read to the children the sequences of digits and letters and the child was required to repeat each sequence in the order in which the items had been presented. In the backward span task, the child heard the same sequences read by the experimenter and was instructed to repeat each sequence in the exact opposite order. The task was interrupted if the child failed to correctly repeat three consecutive sequences. The order of testing was the same for all children: the forward digit span test was administered first, then the backward digit span test, followed by the three language tests [comprehension of (a) OVS sentences; (b) pronouns and reflexives; and (c) relative clauses].

## Results

We analyzed the data using the *lme4* package (Bates et al., 2014) in the R environment (R Development Core Team, 2014). The categorical accuracy data were analyzed with logit mixed models (Jaeger, 2008). The eye-tracking data were analyzed using linear mixed models with empirical logit as dependent variable (Barr, 2008). The eye-gaze plots present the data after having removed the individual differences from the dependent variable, based on the outcome of the linear mixed model. This was done using the *remef* function (Hohenstein and Kliegl, 2013). The plots therefore present the results on which the statistical inferences are based, that is, the ones that are derived from the statistical model. Importantly, in the case of the data presented here, plotting the partial effects yielded patterns qualitatively similar to those of the observed data. This means that removing the individual differences did not alter the general pattern in the data. For each of the eye-gaze plots, a corresponding figure showing the observed data is provided in the online supplementary material, for the sake of comparison. Memory Score (average score on the two span tests) and Language Score (average score on the three language tests) were inserted into the mixed-effects model analysis as continuous covariates, without splitting the group of participants. However, for the sake of presenting the data (either in a plot or in a table), the group was divided into children who scored higher vs. those who scored lower on the tests. This division was done with a median split. Scatterplots showing the individual performance pattern (for both the accuracy and the eye-tracking data) in relation to the average score on the memory and language tests can be found in the online supplementary material. In this section, we report the most relevant results of the analyses. The complete output of each model is listed in the online supplementary material.

The data from six children who did not do the memory and language tests were excluded, so the analysis of the accuracy data is based on 41 children. For two among these, eye-tracking failed due to technical problems during the testing session. Thus, the analysis of the eye-tracking data is based on 39 children. In the eye-tracking data analysis, we excluded 35 trials (2.2% of the total trials available) in which there was more than 50% data loss. The excluded items were distributed across all conditions and several participants. Prior to the analysis, we checked whether the participants performed similarly on trials with the verb *jagen* ‘chase’ and on those with the verb *kitzeln* ‘tickle.’ There was no substantial difference in the performance on trials involving these two actions, neither in terms of response accuracy nor in terms of eye-gaze. Hence, all trials were analyzed together.

### Accuracy

Response accuracy was calculated based on the color named by the participants (Arnon, 2010). Naming the color of the target animal was scored as 1; otherwise as 0. Without taking into account the individual differences of language and memory abilities, children performed on the OR + 1pro condition 97% (SE = 0.03) accurately, on the OR + 2DP condition 47% (SE = 0.02) and on the OR + 3pro condition 44% (SE = 0.03). These accuracy percentages were compared to chance level using one-sample *t*-tests (chance level was set at 0.5 since, although there were three regions of interest in the visual scene, children never named the color of the middle animal, indicating that they never considered it a possible answer). Only performance on the OR + 1pro condition was significantly above chance (*t* = 43.06). On the OR + 2DP and OR + 3pro conditions, performance was at chance (*t* = −0.59 and *t* = −1.16, respectively).

The horizontal dashed line marks the chance level of 0.5.

**FIGURE 2:**
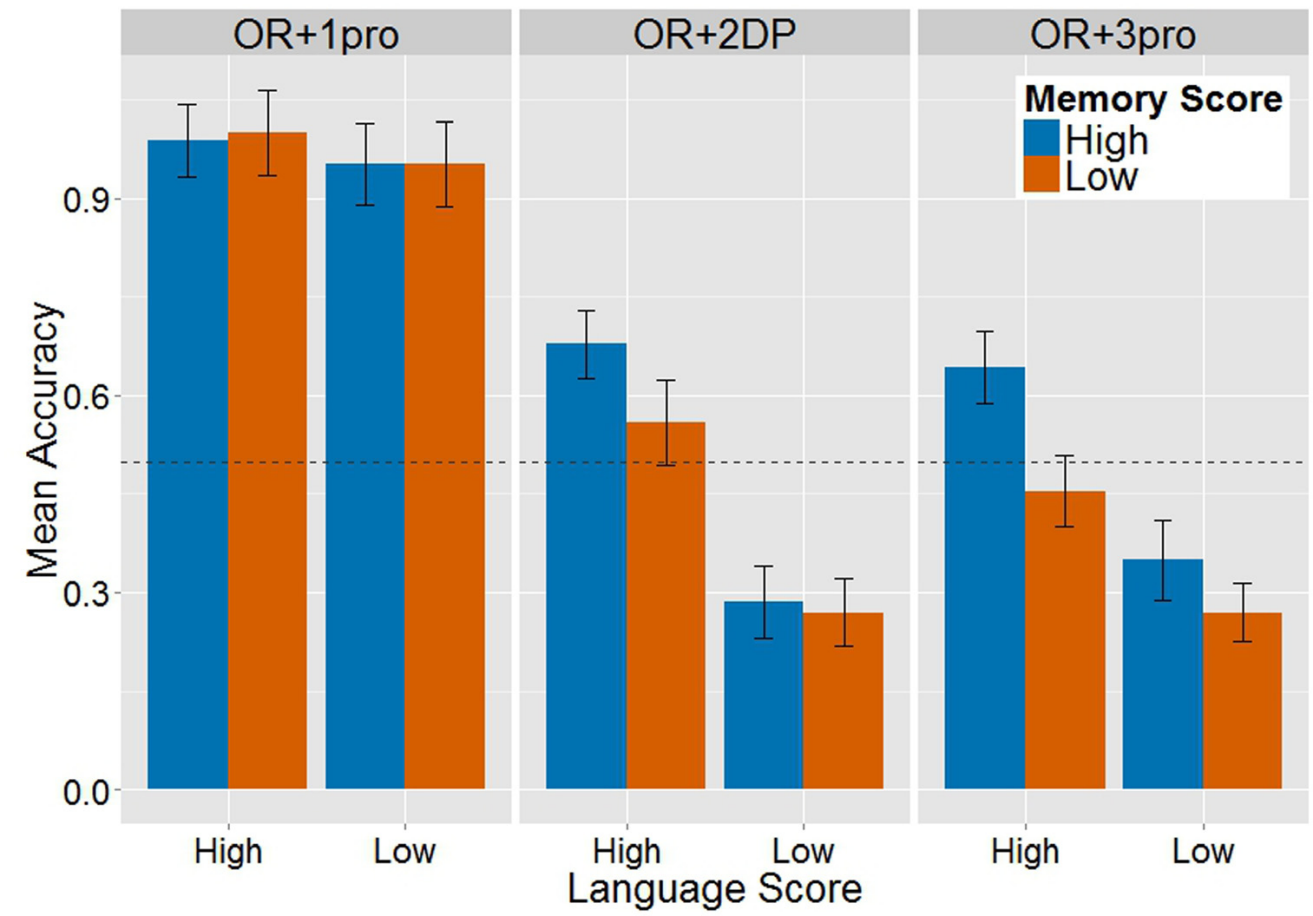
Mean response accuracy (±1 SE) on the three conditions, in relation to children’s scores on the language tests (on the *x*-axis) and on the memory tests (blue = High Score; orange = Low Score).

The results look different when language and memory abilities are considered. **Figure 2** shows the pattern of relation between children’s scores on the language and memory tests, and how it is manifested in their performance on each of the three conditions. The ceiling performance on the OR + 1pro condition was not influenced by language and memory abilities. The pattern that emerges in the OR + 2DP condition is similar to that in the OR + 3pro condition. A lower score on the language tests determined a below-chance performance on these two conditions, whereas a higher score on the language tests determined a more accurate performance on them.

The accuracy data were fit into a logit mixed model, including Condition as fixed factor, Language Score and Memory Score as two continuous covariates (without splitting the participant group) and intercepts for random effects of subjects and items. The OR + 1pro condition was excluded from the analysis to avoid the impact of extreme differences in task performance on the model outcome. All the terms that contain an interaction between Language and Memory were included, since these two covariates did not correlate significantly (*r* = 0.08, *t* = 0.45). A table of correlations between the language measure, the memory measure and response accuracy is provided in the online supplementary material. The main effect Condition was not statistically significant (coef = -0.12, SE = 0.49, *z* = -0.25, *p* = 0.81), confirming that performance on OR + 2DP and OR + 3pro was overall similar. The main effect Language Score was significant (coef = 0.36, SE = 0.16, *z* = 2.26, *p* = 0.02), and so was the interaction Condition by Language Score (coef = -0.31, SE = 0.13, *z* = -2.34, *p* = 0.02). This interaction reflects the fact that, whereas performance on the OR + 2DP and OR + 3pro conditions was the same in children with lower language scores, children with higher language scores were significantly more accurate on OR + 2DP than on OR + 3pro. None of the terms that include Memory Score (main effect Memory and the interactions Condition by Memory, Language by Memory as well as Condition by Language by Memory) was statistically significant. Hence, we see that children’s performance on OR + 2DP and OR + 3pro in the off-line data is modulated by language, but not by memory capacity.

### Eye-Tracking

**FIGURE 3:**
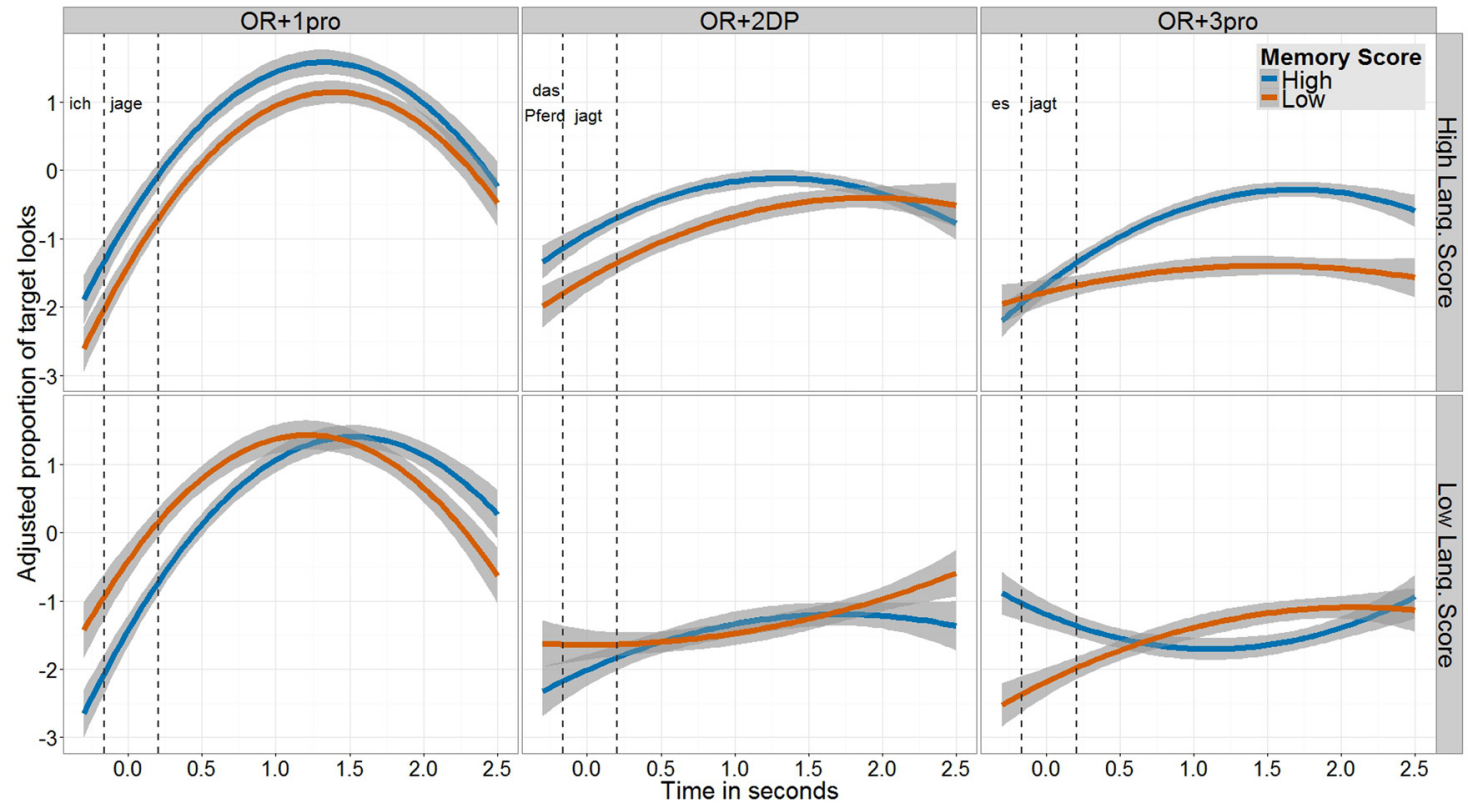
**Proportion of target looks (transformed to empirical logit and adjusted after the removal of individual differences) within the time window relevant for analysis, shown separately for each condition, divided by children’s score on the memory tests (blue line = High Score; orange line = Low Score) and broken by their score on the language tests (top row = High Score; bottom row = Low Score).** On the *x*-axis Time ranges from the offset of the relative pronoun until the end of the 2-s long silence that followed the sentence. Two vertical dashed lines mark the critical chunks in the analysis window: (1) embedded subject DP (*ich* ‘I’; *das Pferd* ‘the horse’; *es* ‘it’); (2) embedded verb (*jage/t* ‘chase/s’); (3) post-sentential silence. The analysis of the eye-gaze data was performed on the entire time window shown in the plot (chunks 1–3).

**Figure 3** shows, for each of the three conditions, the proportion of target looks of children with high and low scores on the memory tests, broken by their scores on the language tests in order to see the relation between the two cognitive measures. The plot shows the data within the relevant time window, defined *a priori* for the analysis, rather than for the entire trial duration. This window starts at the offset of the relative pronoun *den* (plus 200 ms, the average time span necessary for programming and executing an eye movement; Trueswell, 2008). Note that the part that precedes the relative pronoun (*Welche Farbe hat der Hase,…* ‘What color has the bunny…’) is ambiguous about whether the sentence is a SR or an OR. However, based on the unambiguously accusative case-marked relative pronoun, it is already possible (and, indeed, very likely for adult speakers at least) to correctly predict that the sentence will turn out to be an OR. For these reasons, the beginning of the critical time window has been set at the beginning of the critical information in the sentence, that is, after the relative pronoun has been processed. This window ends after the 2-s long silence that followed the test question.

Within this time window, the effects we are interested in might start from the onset of the embedded subject DP onward, while the embedded full DP or pronoun and the verb are processed. Another (perhaps more plausible) possibility is that the effects emerge also in the 2-s long silence following the test sentence. In other words, children might continue to process the structure even after the sentence offset (Brandt-Kobele and Höhle, 2010; Adani and Fritzsche, 2015). Importantly, by including the post-sentential silence in the analysis time window we account for effects that might occur upon processing the verb, which is the very last word in the sentence. This is relevant in the light of studies with adults that predict the effect to occur at the verb, the point in which the filler-gap dependency is resolved (e.g., Gibson, 2000; Gordon et al., 2001, 2002; Warren and Gibson, 2002; Lewis et al., 2006; O’Grady, 2011).

Within the critical time window, which was approximately 2800 ms long, the dependent variable was the proportion of looks to the target figure, calculated as looks to the target animal divided by looks to all the three animals in the visual scene. An accurate processing of the sentence in terms of eye-gaze might be expressed by faster looks to the target (earlier increase in proportion of target looks, or PTL), by more target looks (higher PTL), or by both. Note that, in the analysis procedure adopted here (Barr, 2008), Time is included in the model as a continuous covariate. Therefore, the analysis does not provide information about the specific point in which the effect occurs. For this reason, we will not be able to say how long exactly after the embedded subject DP or the embedded verb have been processed the effect starts. However, the advantage in such an analysis is that the time-related information is obtained in its entirety, without the necessity to cut time into chunks and lose information about the timely course of the gaze pattern. The time-related information is expressed here in the form of significant interactions with the Time covariate. For instance, a significant interaction Condition by Time would mean that, over time (without knowing where exactly during the analyzed window), target looks in one condition increase more than in another condition. For the analysis, each of the pronoun conditions was compared to the baseline condition with two full DPs, using sliding contrast specification (OR + 1pro vs. OR + 2DP vs. OR + 3pro). The plot and analysis of the eye-gaze data include all the trials in the experiment, independently of whether they were answered correctly or incorrectly.

Let us turn to the gaze pattern shown in **Figure 3**. In the OR + 1pro condition, the increase in target looks is faster and the PTL is higher (peaking around 1200 ms into the critical time window) than in the other two conditions, reflecting what we find in the accuracy data. Individual differences in language and memory skills do not appear to affect this pattern. In the OR + 2DP condition, children with a low score on the language tests look less to the target independently of their memory score (lower middle panel in **Figure 3**). Children with a higher language score (upper middle panel) look faster to the target when their memory score is high (culminating at about 1500 ms), as compared to when their memory score is low. These high-language but low-memory children eventually look to the target like their high-memory peers, but at a later point (around 1800 ms). In the OR + 3pro condition, children with a low language score again look less to the target independently of their memory score (lower right panel). However, a clear difference emerges between high-memory and low-memory children when their language score is high (upper right panel). Here, high-memory children look to the target faster and more than their low-memory peers.

Following Barr’s (2008) procedure for the analysis of eye-tracking data in the visual-world paradigm, we performed only the by-subject analysis, aggregating the data across items. This was done due to the relatively small number of items per condition. The proportion of target looks was transformed to an empirical logit and used as the dependent variable in the model. Time, divided into 50 ms long bins, was centered around the point in which target looks started to increase when all conditions are collapsed together, based on a Grand Mean plot. We then fit a linear mixed model including Condition as fixed factor, Time as covariate with linear and quadratic polynomials, Language Score and Memory Score as additional continuous covariates (without group splitting) and an intercept for the random effect of subjects. As in the model for the accuracy data, all the terms that contain an interaction between Language and Memory were included as well, due to the lack of correlation between the two measures. The inclusion of a quadratic term for Time was justified by a comparison to a model with a linear term only (χ^2^ = 726.3, difference in Df = 12, *p* < 0.001).

The main effect Condition was significant for both comparisons, but in opposite directions: PTL in the OR + 1pro condition were significantly greater than those in the OR + 2DP condition (coef = −0.82, SE = 0.03, *t* = −30.88); PTL in the OR + 2DP condition were significantly greater than those in the OR + 3pro condition (coef = −0.25, SE = 0.03, *t* = −9.46). These effects mean that children looked to the target in OR + 1pro trials overall longer than in OR + 2DP trials, and in these longer than in OR + 3pro trials. The former effect reflects what we find in the accuracy data, but the advantage of OR + 2DP over OR + 3pro in terms of eye-gaze is absent in the accuracy data. Both the main effect of Language (coef = 0.06, SE = 0.03, *t* = 1.98) and the main effect of Memory (coef = 0.09, SE = 0.05, *t* = 1.87) were only marginally significant. Also the interaction Language by Memory was not statistically significant (coef = 0.07, SE = 0.04, *t* = 1.73). Most importantly, all the four-way interactions were significant. For the comparison OR + 1pro vs. OR + 2DP, the interaction Time by Condition by Language by Memory was significant (for the quadratic term of Time: coef = 3.88, SE = 1.82, *t* = 2.13). This effect reflects the pattern observed in the two middle and the two left panels of **Figure 3**. No individual differences in language and memory emerge in the performance on the OR + 1pro condition, whereas differences do emerge in the OR + 2DP condition depending on language and memory scores. Also for the comparison OR + 2DP vs. OR + 3pro, the interaction Time by Condition by Language by Memory was significant (for the linear term of Time: coef = 8.41, SE = 1.80, *t* = 4.66; for the quadratic term of Time: coef = −6.39, SE = 1.76, *t* = −3.63). This effect reflects what we see in the two middle and the two right panels of **Figure 3**. When language score is low, the gaze pattern in the two conditions is the same independently of the memory score. But when language score is high, the differences between high-memory and low-memory children are more pronounced in the OR + 3pro condition than in the OR + 2DP condition: only in the latter the low-memory children eventually look to the target like their high-memory peers, albeit later.

### Looks to Distractor

**FIGURE 4:**
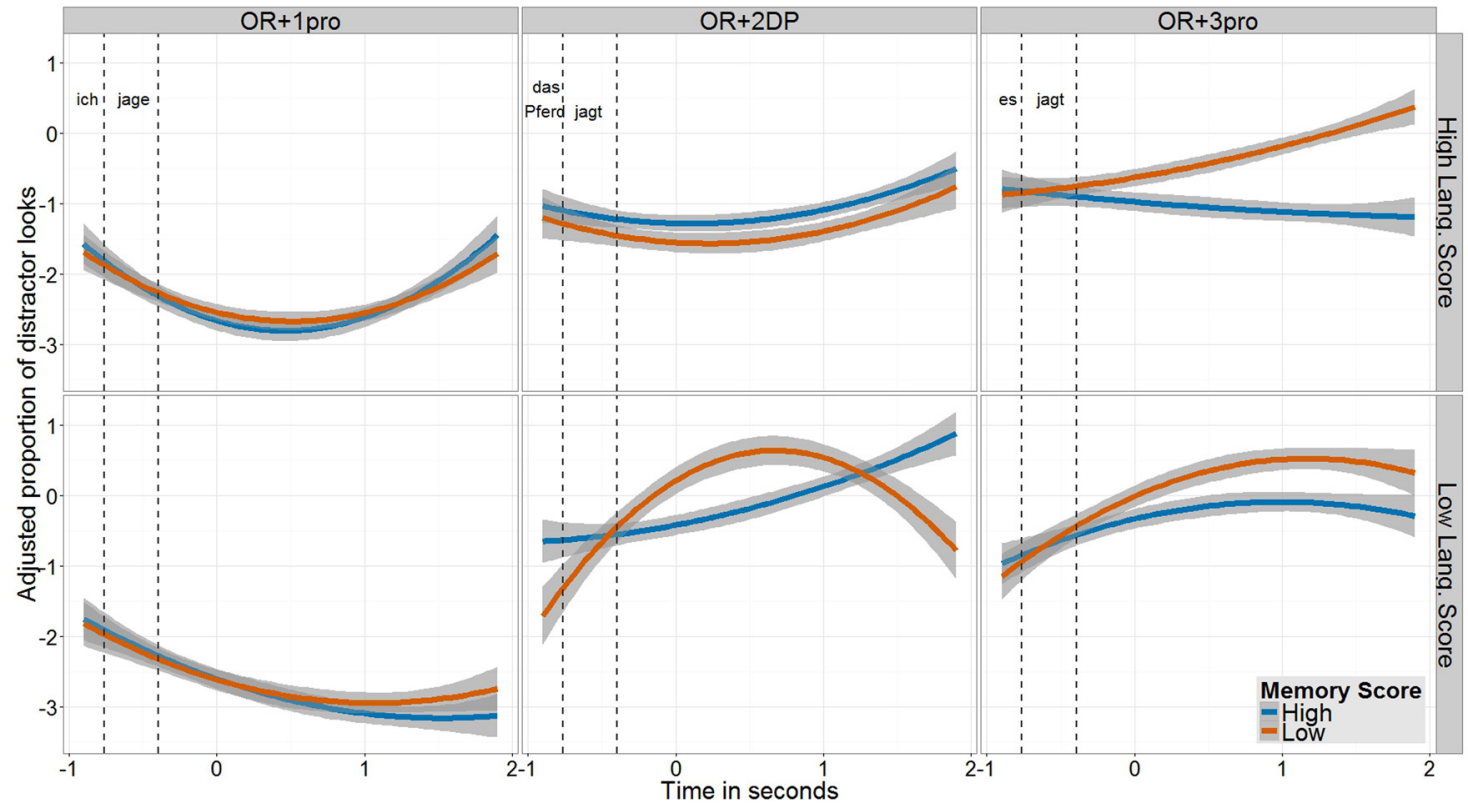
**Proportion of looks to the distractor figure (transformed to empirical logit and adjusted after the removal of individual differences) within the time window relevant for analysis, shown separately for each condition, divided by children’s score on the memory tests (blue line = High Score; orange line = Low Score) and broken by their score on the language tests (top row = High Score; bottom row = Low Score)**. On the *x*-axis Time ranges from the offset of the relative pronoun until the end of the 2-s long silence that followed the sentence. Two vertical dashed lines mark the critical chunks in the analysis window: (1) embedded subject DP (*ich* ‘I’; *das Pferd* ‘the horse’; *es* ‘it’); (2) embedded verb (*jage/t* ‘chase/s’); (3) post-sentential silence.

Before discussing the results, let us examine the pattern of children’s looks to the distractor animal. Recall that, in their off-line responses on incorrect trials, children named the color of the distractor animal, never that of the middle animal. **Figure 4** shows, for each of the three conditions, the proportion of distractor looks in children with high and low scores on the memory tests, broken by their language scores (again, we plot here the partial effects; the corresponding plot showing the observed data is provided in the online supplementary material). As expected, and reflecting children’s off-line responses, on the OR + 1pro condition their looks to the distractor are very low. By contrast, on the OR + 2DP and OR + 3pro conditions, the proportion of distractor looks throughout the critical time window is very high, mostly for children with lower memory scores. That is, children’s errors were expressed by their systematic (off-line as well as on-line) interpretation of the OR as a SR, treating the DP head as the subject rather than the object of the embedded clause. This pattern of error is typically found in studies on children’s comprehension of relative clauses.

## Discussion

The aim of the study was to test the effects of various pronoun types on children’s processing of ORs. We took as reference condition ORs with a full DP head and an embedded full DP subject, which are typically hard for children, and manipulated the embedded subject using personal pronouns. The three OR types were structured with a masculine noun as DP head, which had the advantage of facilitating, at least potentially, children’s comprehension. This was achievable due to the possibility to recognize the sentence as an OR rather early in the sentence, upon processing the accusative case-marking on the relative pronoun (*den*). There is evidence from previous studies on relative clause comprehension in German (Arosio et al., 2012) that children are facilitated when the relative clause (whether a SR or an OR) is disambiguated by case (as in our stimuli), as compared to when it is disambiguated by a singular or plural number-marking on the embedded verb (in our stimuli, the verb was always marked with singular). Another characteristic of the three conditions we tested is that they differ with respect to the referring expression in the embedded subject position–full DP, 1st- or 3rd-person pronoun. We therefore expect these referring expressions to trigger effects in task performance, if their referential properties play a role in determining OR processing. The initial prediction, as made by Friedmann et al. (2009) and by other accounts, is that ORs with embedded pronominal subjects are more accurately comprehended than ORs with two full DPs, independently of the pronoun type. Our findings support this prediction only partially.

First, we find that children are more accurate on ORs with an embedded 1st-person pronoun than ORs with two full DPs, both in terms of off-line accuracy and in terms of on-line eye-gaze, where we find more target looks in the OR + 1pro than in the OR + 2DP condition. This finding supports the initial prediction. It is also in line with other studies, both with children and with adults, showing that a 1st- or 2nd-person pronoun in the embedded subject position makes the OR easier to process (Gordon et al., 2001; Warren and Gibson, 2002, 2005; Arnon, 2010).

We also find that ORs with 1st-person pronoun are more accurately processed (again, both off-line and on-line) than ORs with 3rd-person pronoun. This result is not in line with the RM account, since the prediction is that different pronoun types in the embedded subject position facilitate ORs to an equal extent. The reason is that in both cases the full DP head, which contains the [+NP] feature, crosses an intervening pronoun, a constituent that lacks the [+NP] feature. This result appears to disagree also with other accounts that predict facilitated performance on ORs with an embedded pronoun, independently of the pronoun type (e.g., Gordon et al., 2001; Lewis et al., 2006). The pronoun asymmetry suggests that defining the (dis)similarity between the DP head and the embedded subject DP only in terms of ‘lexical restriction,’ that is, in terms of a full DP vs. a personal pronoun, is not sufficient. This pronoun asymmetry is in line, however, with theoretical accounts on referential properties of pronouns (Heim, 1991; Recanati, 1993; Erteschik-Shir, 1997; Ariel, 2001; Legendre and Smolensky, 2012) as well as with previous experimental studies with adults. Both Warren and Gibson (2002) and Carminati (2005) found that the presence of a 1st-person pronoun facilitates adults’ sentence processing more than the presence of a 3rd-person pronoun. These studies explain such an asymmetry in terms of the different referential properties of the pronouns. Since discourse referents of 1st-person pronouns are accessed directly, these pronouns are less costly for processing than 3rd-person pronouns, which need to be resolved via an antecedent (in the sentential or extra-sentential context), before the discourse referent of the pronoun is accessed. This is also the case in the present study: the discourse referent of the 3rd-person pronoun is accessed only after the pronoun has been resolved via an antecedent, which had to be retrieved from the linguistic context provided in the preamble video before the trial. Hence, the presence of the pronoun in itself does not necessarily facilitate OR processing. It seems that only pronouns that relate to their discourse referents directly, like 1st-person pronouns, do so ^6^. The facilitation found by Friedmann et al. (2009) with Hebrew ORs containing an embedded arbitrary *pro* subject (example 6 in the *Introduction*) can be explained on similar terms. The Hebrew arbitrary *pro* is used when the agent of the action remains unspecified. It might well be that the facilitation was due to the discourse properties of *pro*–the fact that it does not relate to any specific discourse referent, thus reducing processing cost–rather than to its property of lacking the [+NP] feature, as suggested by the authors.

A third pattern, that emerges in the eye-gaze data, is that ORs with a 3rd-person pronoun are actually harder for children than ORs with two full DPs. This finding is not in line with the prediction that any kind of pronoun in the embedded subject position facilitates OR comprehension (e.g., Gordon et al., 2001; Friedmann et al., 2009; Rizzi, 2013). It can be explained, again, if the referential properties of the referring expressions are taken into account. A 3rd-person pronoun can be interpreted only after it has been related to an antecedent, which needs to be located and retrieved from the linguistic or extra-linguistic context. This is not the case with a full DP, whose discourse referent is derived from its lexical meaning and accessed directly. Note that, just like in an OR with a 1st-person pronoun, also in an OR with 3rd-person pronoun the DP head crosses an intervening pronoun. The fact that the former condition is easier than the latter, compared to the baseline with two full DPs, supports further the claim that the presence of the pronoun on its own cannot account for children’s performance. Rather, the type of pronoun–and more precisely, the referential properties of that pronoun–appear to play a major role in facilitating or not facilitating the processing of the OR.

Interestingly, Goodluck (2005, 2010) managed to separate intervention locality effects from complex discourse accessibility operations. Goodluck (2005) manipulated the discourse accessibility operation in object-extracted wh-questions by making it more demanding (*Which lion did the zebra kiss?*) or less demanding (*Which animal did the zebra kiss?*). Crucially, in both cases, the intervention locality effect was present (in both sentences, both the moved object DP and the intervening subject DP are lexically restricted). The fact that children were more accurate on the *which-animal* question than on the *which-lion* led the author to conclude that discourse accessibility determines children’s performance on the structure independently of the syntactic complexity. This is reminiscent of what we find in the two pronoun conditions. Both in OR+1pro and in OR + 3pro, the (reduced) syntactic complexity is kept constant due to the embedded pronoun. Therefore, children’ higher accuracy rate on OR + 1pro than on OR + 3pro is likely due to the different referential properties of the pronouns. In other words, the direct discourse accessibility in the case of the 1st-person pronoun makes this condition easier than the 3rd-person pronoun condition, in which discourse accessibility is indirect and therefore more demanding.

Note that the advantage of the OR + 2DP condition over OR + 3pro, in terms of main effect, is found only in the on-line eye-gaze data. An even more crucial finding is that the effects of memory only emerge in the on-line data, whereas they remain hidden when looking at the off-line accuracy data. These findings join a growing body of studies that show that children’s performance sometimes appears different when tested by means of explicit or implicit responses. Specifically, measures of implicit processing (such as eye-tracking) often suggest that children accurately parse ORs even though their explicit performance on the same ORs remains poor (Adani and Fritzsche, 2015; see also discussion in Brandt-Kobele and Höhle, 2010). In the present study we show that children looked faster or longer to the target figure in conditions that they processed more accurately than in conditions that were harder for them. In other words, when children correctly processed a sentence their attention on the target figure was more stable in comparison to harder sentences.

These eye-gaze effects were found within the 2800 ms long time window defined *a priori* for the analysis. A widespread assumption, supported by evidence from on-line processing studies with adults, is that such effects occur upon processing the embedded verb of an OR, the site in which the filler-gap dependency is resolved (e.g., Gibson, 2000; Gordon et al., 2001, 2002; Warren and Gibson, 2002; Lewis et al., 2006; O’Grady, 2011). Although Friedmann et al. (2009) do not make specific predictions regarding the exact point in which intervention effects occur, it seems they do so in subsequent work (Belletti et al., 2012), suggesting that intervention effects are detectable only when the two relevant DPs (the head noun and the embedded subject in an OR) are similar in terms of morphological features that are overtly marked on the embedded verb. Hence, it seems that also according to the RM account intervention effects in ORs are expected to occur at the embedded verb. This idea is entertained also in Franck et al. (2015).

Analyzing the eye-gaze data in the entire time window from the offset of the relative pronoun until the end of the post-sentential silence does not allow the detection of time-locked effects. Nevertheless, it had several motivations and some evident advantages. First, the part of the sentence that precedes the relative pronoun, which was equal in the three conditions, is not informative enough to guide the participants toward the identification of the relevant referent. We therefore do not expect any gaze pattern prior to hearing the relative pronoun to be driven by the linguistic input. Second, processing the unambiguously accusative case-marked relative pronoun is virtually enough to be able to identify the sentence as an OR and thus the correct referent. Even though we do not expect to find evidence for such rapid processing in 5-year-olds, the crucial point is that the relative pronoun is the first informative point in the sentence. Third, young children might be slow in processing the OR, and effects stemming from their eye-gaze might well emerge after the critical information has been processed. Several visual-world studies have even found effects occurring after the sentence ended (e.g., Brandt-Kobele and Höhle, 2010; Adani and Fritzsche, 2015). Crucially, the embedded verb in our stimuli is the last word in the sentence. Thus, post-sentential effects might be driven (also) by the filler-gap dependency resolution at the verb, as predicted, for instance, by Gibson (2000), Gordon et al. (2001, 2002), Warren and Gibson (2002), Lewis et al. (2006), O’Grady (2011) and other account. Finally, following Barr’s (2008) analysis procedure, the inclusion of Time as a continuous covariate appears to be more appropriate in a linear mixed-effects model analysis. The main reason is that the effect of time (the change in gaze pattern throughout the duration of the trial) is captured in its entirety, whereas by cutting it into chunks some information about the time course of the gaze pattern is lost.

Concerning language and memory abilities, we have looked at the role of children’s memory capacity in their OR processing and at its relation to the role of their language skills. The goal was to test whether effects which are due to language and memory depend on each other or not and, if they do, in what manner. We had previously shown that, on the two harder conditions (OR + 2DP and OR + 3pro), children with stronger language abilities are significantly more accurate than children with weaker language skills (Haendler et al., 2015). Given the linguistic material used in the three administered subtests, we reasoned that stronger language or grammatical skills meant a stronger ability to compute movement-derived structures (subtests on sentences with canonical and non-canonical word order) and a stronger ability in discourse accessibility operations (subtest on reflexives and pronouns). It is therefore not surprising that children who had a higher average score on these tests were more accurate on ORs that were more difficult in terms of computing the syntactic movement (OR + 2DP) and on ORs that were more difficult in terms of discourse accessibility (OR + 3pro). On the OR + 1pro condition, in which both the computation of the syntactic movement and discourse accessibility are facilitated, all children were accurate independently of their score on the grammatical tests.

In the present study, adding memory abilities to the picture reveals a more fine-grained pattern in the effects of language skills previously found. The analysis shows that language and memory have independent, additive effects that vary in relation to the experimental conditions. Children are most accurate on the OR + 1pro condition, but neither their response accuracy nor their eye-gaze are influenced by individual differences in language and memory abilities. Individual differences in language and memory do affect, however, performance on the OR + 2DP and OR + 3pro conditions, but the effects of memory are observable only in the eye-gaze data, as mentioned earlier. Whether children with weaker grammatical skills have stronger or weaker memory does not seem to affect their performance substantially. By contrast, the gaze pattern of children with stronger grammatical skills clearly changes depending on their memory capacity. In the OR + 2DP condition, low-memory (and high-language) children look to the target like their high-memory peers, but later, suggesting an accurate albeit delayed processing of the sentence. In the OR + 3pro condition, low-memory (and high-language) children look to the target less than their high-memory peers up to the end of the trial, showing no evidence of correct processing of the sentence. **Table 1** summarizes these findings in a schematic way.

**Table 1:** A summary of the cases in which we find evidence for accurate processing (in terms of on-line target looks) of the different conditions, depending on language, and memory abilities.

		OR + 1pro	OR + 2DP	OR + 3pro
High-language	High-memory	YES	YES	YES
	Low-memory	YES	YES, but late	NO
Low-language	High-memory	YES	NO	NO
	Low-memory	YES	NO	NO

*YES, there is such evidence; NO, there is no such evidence.*

To account for these results, we will now explain what might cause the qualitative differences among the conditions and how language and memory abilities might play a role in creating the effects we find. The three conditions are similar in their syntactic structure, in the sense that they are all ORs in which the DP head moves from the embedded object position. Processing this movement, and resolving the filler-gap dependency, is assumed to be facilitated in the two pronoun conditions. According to the RM account, the syntactic complexity of OR + 1pro and OR + 3pro is reduced due to the attenuation of the intervention locality effect, since the full DP head crosses an intervening pronoun rather than another full DP (Friedmann et al., 2009; Rizzi, 2013). The syntactic complexity of ORs with pronouns is reduced also from the perspective of the integration cost metric account (Gibson, 1998, 2000; Warren and Gibson, 2002, 2005) and according to the similarity-based and cue-based interference approach (Gordon et al., 2001, 2002, 2004; Lewis and Vasishth, 2005; Lewis et al., 2006; van Dyke and McElree, 2006, 2011). All these accounts argue that facilitated processing of ORs with embedded pronouns is due to reduced burden on memory resources (see also Sheppard et al., 2015). The three conditions differ, however, with respect to the referring expression in the embedded subject position: these referring expressions require different levels of processing cost in terms of discourse accessibility. The 1st-person pronoun and the full DP relate to their discourse referents directly, deriving them from their lexical meanings, whereas the 3rd-person pronoun relates to its discourse referent indirectly, deriving it from the meaning of the antecedent to which it relates. This implies that referring expressions (such as 1st-person pronouns and full DPs) whose discourse referent is accessed directly overload memory resources less than referring expressions (such as 3rd-person pronouns) whose discourse referent has to be retrieved from the previously encoded context (Warren and Gibson, 2002; van Rij et al., 2013).

These syntactic and discourse characteristics of the conditions appear to explain the pattern we find in the data. In particular, they might account for the role of memory capacity and its additive effects to those of language skills. Language skills, as defined by the average score on the three language tests, appear to be the underlying constraint on children’s performance. If children score low on these tests–in other words, if they are less proficient in processing movement-derived structures and in accessing discourse (these are the two relevant operations assessed by the language tests, as we have seen)–then we find no evidence for accurate processing of the two conditions that are hard either due to syntactic movement (OR + 2DP, in which a full DP moves over another full DP) or due to discourse accessibility (OR + 3pro, in which accessing the discourse referent of the 3rd-person pronoun is more demanding). It seems that, in the case of low-language children, some basic grammatical skills are weaker and therefore their memory capacity does not make any difference. Not surprisingly, even low-language children succeed on the OR + 1pro condition, which is less demanding both in terms of its syntactic movement and in terms of discourse accessibility. But also here memory capacity does not make any difference: this condition is equally easy for all children independently of their memory skills. What happens in children who score high on the three language tests? Just like their low-language peers, they perform at ceiling on the easiest OR + 1pro condition, independently of their memory capacity. A different pattern, modulated by memory, emerges in the two harder conditions (OR + 2DP and OR + 3pro). In OR + 2DP, high-memory children correctly process the structure, whereas low-memory children do so as well, but rather late. In OR + 3pro, there is evidence that only high-memory children correctly process the structure, whereas low-memory children are substantially less accurate.

Thus, memory capacity appears to be crucial when discourse accessibility is demanding (as when 3rd-person pronouns need to be resolved), but only if general linguistic abilities, such as computing syntactic movements and accessing discourse referents of pronouns and reflexives, are sufficiently strong. In the OR + 2DP condition, in which retrieving the referent of a full DP is less costly, even low-memory children eventually look to the target, although later than their high-memory peers. In the OR + 3pro condition, in which the retrieval of the referent of the 3rd-person pronoun is more costly, low-memory children do not catch up with their high-memory peers and there is no evidence that they accurately process the structure.

Our findings resemble, at least partly, those of Warren and Gibson (2002), who elaborate on the idea that memory resources are crucial for processing structures that require both filler-gap dependency resolution and accessing discourse referents of various referring expressions. These authors found the same asymmetry between 1st-person pronouns and 3rd-person pronouns, with the former facilitating OR processing more than the latter, an asymmetry which is explained in the light of Gibson’s (1998, 2000) integration cost metric. According to the authors, the processing cost of a certain structure increases with the number of discourse referents that intervene between the filler and the gap site in which it is integrated. The reason is that each of the intervening discourse referents has to be integrated as well, thus reducing the memory resources available to process the structure. When one of the intervening discourse referents is a 1st-person pronoun, whose integration is done straightforwardly, the available memory resources are less burdened than in the case in which the intervening constituent is a 3rd-person pronoun, whose integration is more costly. Note, however, that in Warren and Gibson (2002) adults judged ORs with an embedded 3rd-person pronoun as less complex than ORs with two full DPs. This pattern is unlike what we find with children. In the present study, OR + 3pro appears to be the condition on which memory has the strongest impact. Given that children’s memory abilities are underdeveloped, compared to adults,’ it is not surprising that children with weaker memory skills struggle while processing ORs with an embedded 3rd-person pronoun, even if their ability to perform on the language tests we used is already strong.

## Conclusion

Our data support only in part a purely syntax-based account such as Friedmann et al.’s (2009), or the similarity-/cue-based interference accounts of relative clause processing. While we do find that an embedded 1st-person pronoun facilitates OR processing, we also find that an embedded 3rd-person pronoun does not. It appears that OR processing is constrained not only by the syntactic complexity of the structure, but also by the referential properties of the involved constituents. Both require memory resources and might thus determine difficulties in processing the OR, as has been suggested for adults. The results suggest that both language and memory abilities play a role in modulating these syntactic and discourse accessibility constraints, and that they do so in an independent, additive fashion.

## Conflict of Interest Statement

The authors declare that the research was conducted in the absence of any commercial or financial relationships that could be construed as a potential conflict of interest.

## Footnotes

1 The RM principle was first developed to explain intervention locality effects in extraction from weak islands (Rizzi, 1990). The approach was later extended to explain intervention effects in ORs, assuming a structural proximity between the latter and the original island phenomena (Grillo, 2005, 2009; Friedmann et al., 2009; Rizzi, 2013).

2 We use the term ‘referring expression’ to mean any linguistic form that relates to some discourse referent. This term thus includes both definite noun phrases (full DPs) and pronouns (see Fukumura and van Gompel, 2012; Serratrice, 2013).

3 Some studies have tested children’s comprehension of intra-sentential anaphora. These are sentences in which the referent of the pronoun is inside the same sentence in which the pronoun appears (e.g., Sekerina et al., 2004; van Rij et al., 2010; Clackson et al., 2011). Here we concentrate only on extra-sentential anaphora, where the referent of the pronoun is in the extra-sentential or extra-linguistic (visual) context. This is the relevant case for the present study.

4 In addition to these three conditions, we also tested a fourth condition in which the head noun was a demonstrative pronoun and the embedded subject was a full DP (*Welche Farbe hat der, den das Pferd jagt?* ‘What color has that (the one) that the horse is chasing?’). The predictions regarding this condition are not straightforward, since existing literature is not explicit about whether such a demonstrative bears the [+NP] feature or not. Moreover, unlike this condition, all the others differed minimally by the referring expression in the embedded subject position. Upon suggestion from the two reviewers, we will neither present nor discuss the data from this condition.

5 To be sure, we performed all the analyses after excluding the four children who scored lower than 50% on the test for comprehension of OVS sentences. The results were qualitatively similar to those of the analysis in which these children are included. We therefore report the results from the analysis that includes all children.

6 Recall that the middle animal in the visual scenes accompanying the OR + 1pro condition was always the dog, the narrator. One reviewer pointed out that children’s high performance on this condition might reflect their familiarity with this animal, rather than the effect caused by the pronoun itself. We have already addressed this issue in a follow-up study, yet to be published. Using similar material and methodology, we tested children on different types of relatives (SRs and ORs), in which the figure of the narrator appeared in various experimental conditions and in some fillers. In this setup, it was impossible to anticipate the type of sentence based on the visual presence of the narrator. Importantly, the results show that the 1st-person pronoun advantage over the 3rd-person pronoun persists, similarly to what we find in the present study.
