# Supplementary material for: Discourse accessibility constraints in children’s processing of object relative clauses
Source: Front Psychol. 2015 Jun 23;6:860. doi: 10.3389/fpsyg.2015.00860 (PMC4477058; doi:10.3389/fpsyg.2015.00860)
Supplement: Supplementary file 2 [file Data_Sheet_1.PDF]

Supplementary Material

Discourse accessibility constraints in children's processing of object relative clauses

Yair Haendler[1,*], Reinhold Kliegl[2], Flavia Adani[3]

[1]Department of Linguistics, University of Potsdam, Potsdam, Germany
[2] Department of Psychology, University of Potsdam, Potsdam, Germany

*** Correspondence:** Yair Haendler, Department of Linguistics, University of Potsdam, Karl-Liebknecht-Strasse 24-25, 14476, Potsdam, Germany.
yair.haendler@uni-potsdam.de

1. Full item list

<u>Fillers</u>

1. Welche Farbe hat der Bär mit dem Hut?
   What color has the bear with the hat?

2. Welche Farbe hat der Löwe mit der Brille?
   What color is the lion with the glasses?

3. Welche Farbe hat der Affe mit dem Herzen?
   What color has the monkey with the heart?

4. Welche Farbe hat der Hase mit der Blume?
   What color has the bunny with the flower?

5. Welche Farbe hat der Bär mit der Brille?
   What color has the bear with the glasses?

6. Welche Farbe hat der Löwe mit dem Hut?
   What color has the lion with the hat?

7. Welche Farbe hat der Affe mit der Blume?
   What color has the monkey with the flower?

8. Welche Farbe hat der Hase mit dem Herzen?
   What color has the bunny with the heart?

9. Welche Farbe hat der Bär mit der Blume?
   What color has the bear with the flower?

10. Welche Farbe hat der Löwe mit dem Herzen?
    What color has the lion with the heart?

11. Welche Farbe hat der Affe mit der Brille?
    What color has the monkey with the glasses?

12. Welche Farbe hat der Hase mit dem Hut?
    What color has the bunny with the hat?

<u>OR+2DP</u>

1. Welche Farbe hat der Bär, den das Kamel kitzelt?
   What color has the bear who the camel tickles?

2. Welche Farbe hat der Löwe, den das Zebra jagt?
   What color has the lion who the zebra chases?

3. Welche Farbe hat der Affe, den das Schaf kitzelt?
   What color has the monkey who the sheep tickles?

4. Welche Farbe hat der Bär, den die Ente jagt?
   What color has the bear who the duck chases?

5. Welche Farbe hat der Löwe, den die Maus kitzelt?
   What color has the lion who the mouse tickles?

6. Welche Farbe hat der Affe, den die Katze jagt?
   What color has the monkey who the cat chases?

7. Welche Farbe hat der Hase, den das Pferd kitzelt?
   What color has the bunny who the horse tickles?

<u>OR+1pro</u>

1. Welche Farbe hat der Bär, den ich jage?
   What color has the bear who I chase?

2. Welche Farbe hat der Löwe, den ich kitzle?
   What color has the lion who I tickle?

3. Welche Farbe hat der Affe, den ich jage?

What color has the monkey who I chase?

4. Welche Farbe hat der Bär, den ich kitzle?
   What color has the bear who I tickle?

5. Welche Farbe hat der Löwe, den ich jage?
   What color has the lion who I chase?

6. Welche Farbe hat der Affe, den ich kitzle?
   What color has the monkey who I tickle?

7. Welche Farbe hat der Hase, den ich jage?
   What color has the bunny who I chase?

<u>OR+3pro</u>

1. Welche Farbe hat der Bär, den es jagt?
   What color has the bear who it chases?

2. Welche Farbe hat der Löwe, den es kitzelt?
   What color has the lion who it tickles?

3. Welche Farbe hat der Affe, den es jagt?
   What color has the monkey who it chases?

4. Welche Farbe hat der Bär, den sie kitzelt?
   What color has the bear who she tickles?

5. Welche Farbe hat der Löwe, den sie jagt?
   What color has the lion who she chases?

6. Welche Farbe hat der Affe, den sie kitzelt?
   What color has the monkey who she tickles?

7. Welche Farbe hat der Hase, den es jagt?
   What color has the bunny who it chases?

<u>OR+dem</u>

1. Welche Farbe hat der, den das Kamel kitzelt?
   What color has DEM who the camel tickles?

2. Welche Farbe hat der, den das Zebra jagt?
   What color has DEM who the zebra chases?

3. Welche Farbe hat der, den das Schaf kitzelt?
   What color has DEM who the sheep tickles?

4. Welche Farbe hat der, den die Ente jagt?
   What color has DEM who the duck chases?

5. Welche Farbe hat der, den die Maus kitzelt?
   What color has DEM who the mouse tickles?

6. Welche Farbe hat der, den die Katze jagt?
   What color has DEM who the cat chases?

7. Welche Farbe hat der, den das Pferd kitzelt?
   What color has DEM who the horse tickles?

2. Scatterplots showing the relation between individual performance on the language test for comprehension of OVS sentences (TSVK, subtest 3; Siegmüller et al., 2010) and the overall performance in the experiment.

Panel A: Proportion of response accuracy is plotted on the y-axis.

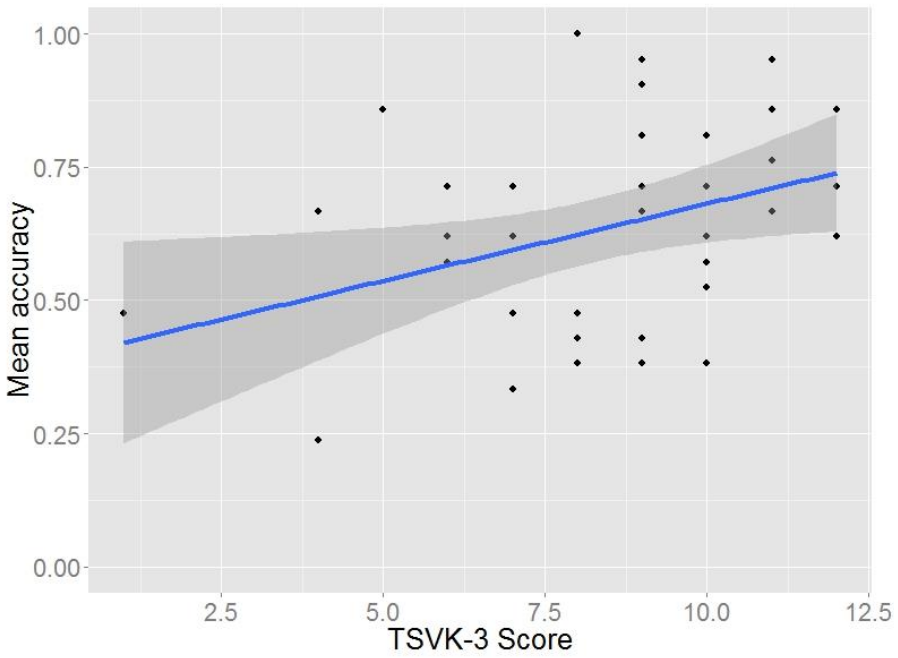

Panel B: Proportion of target looks is plotted on the y-axis.

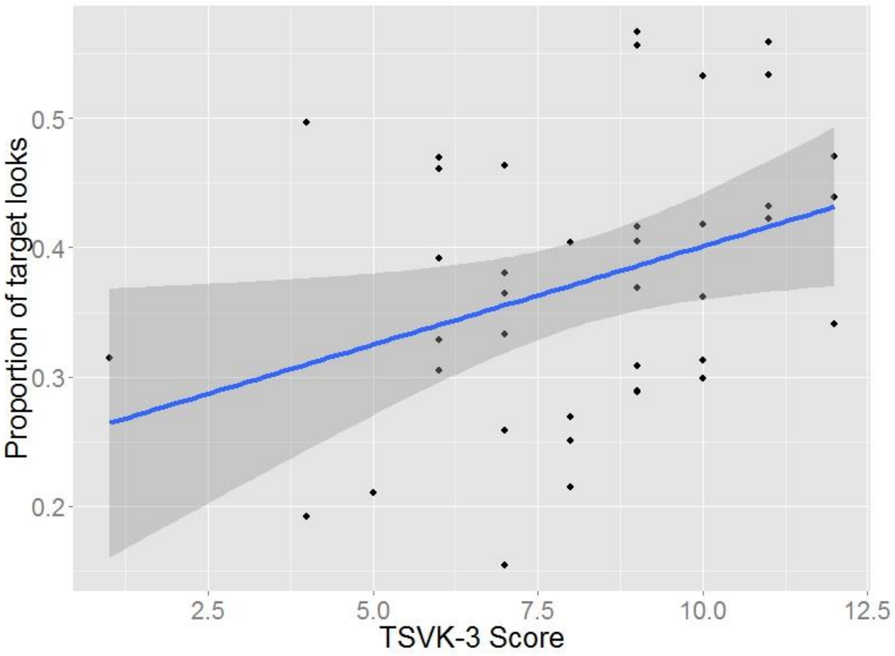

3. An example of preamble text and test sentence for each of the conditions.

Condition	Preamble text	Test sentence
**Fillers**	Hier sind zwei Bären. Der eine Bär ist lila und der andere Bär ist gelb. Und hier ist ihr Freund, das Kamel. Das Kamel ist grün.<br><br>Here are two bears. One bear is purple and the other bear is yellow. And here is their friend, the camel. The camel is green.	Guck mal!<br>Welche Farbe hat der Bär mit dem Hut?<br><br>Look!<br>What color is the bear with the hat?
**OR+2DP**	Hier sind zwei Löwen. Der eine Löwe ist rot und der andere Löwe ist gelb. Und hier ist ihr Freund, das Zebra. Das Zebra ist blau.<br><br>Here are two lions. One lion is red and the other lion is yellow. And here is their friend, the zebra. The zebra is blue.	Guck mal!<br>Welche Farbe hat der Löwe, den das Zebra jagt?<br><br>Look!<br>What color is the lion that the zebra chases?

**OR+1pro**	Hier sind zwei Affen. Der eine Affe ist gelb und der andere Affe ist rot. Und ich bin auch da. Hier bin ich blau.<br><br>Here are two monkeys. One monkey is yellow and the other monkey is red. And I'm also here. Here I'm blue.	Guck mal!<br>Welche Farbe hat der Affe, den ich kitzle?<br><br>Look!<br>What color is the monkey that I tickle?
**OR+3pro**	Hier sind zwei Bären. Der eine Bär ist blau und der andere Bär ist gelb. Und hier ist ihre Freundin, die Ente. Die ENTE ist pink.<br><br>Here are two bears. One bear is blue and the other bear is yellow. And here is their friend, the duck. The DUCK is pink.	Guck mal!<br>Welche Farbe hat der Bär, den sie kitzelt?<br><br>Look!<br>What color is the bear that she tickles?
**OR+dem**	Hier ist ein pinkes Schaf. Und hier sind seine Freunde, die Affen. Der EINE Affe ist gelb, und der ANDERE Affe ist blau.<br><br>Here is a pink sheep. And here are its two friends, the monkeys. ONE monkey is yellow, and the OTHER monkey is blue.	Guck mal!<br>Welche Farbe hat der, den das Schaf kitzelt?<br><br>Look!<br>What color is the one that the sheep tickles?

4. Scatterplots showing the individual performance on the language tests and its relation to overall performance in the experiment (language score is calculated as average score on the three subtests 3, 5 & 6 from TSVK, Siegmüller et al., 2010).

Panel A: Proportion of response accuracy is plotted on the y-axis.

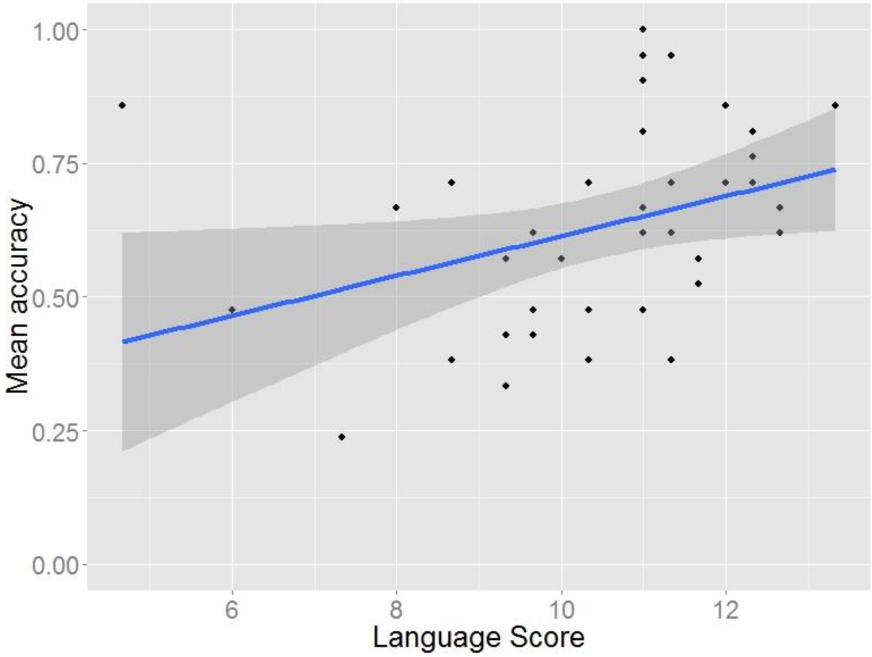

Panel B: Proportion of target looks is plotted on the y-axis.

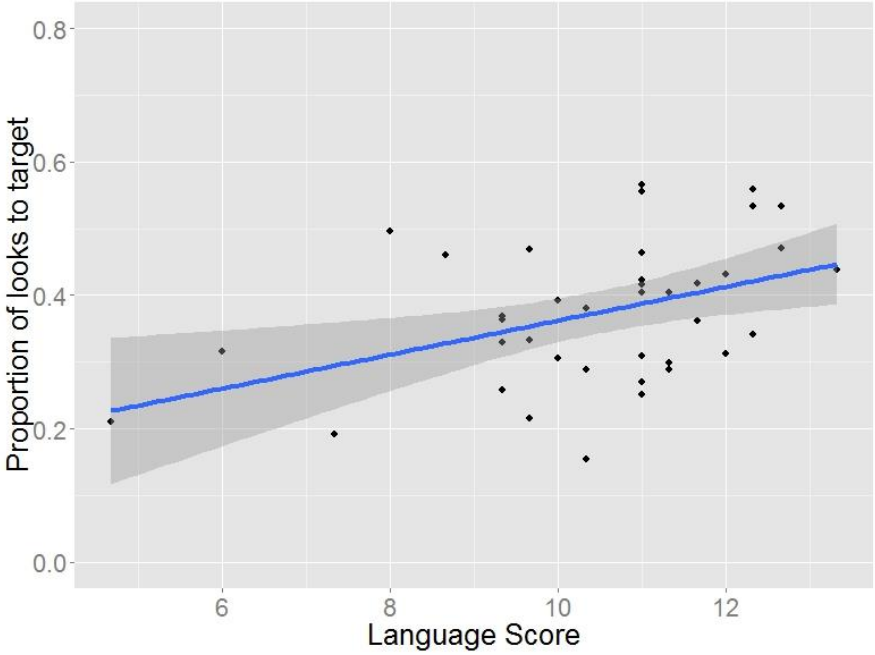

5. Scatterplots showing the individual performance on the language tests and its relation to overall performance in the experiment (language score is calculated as average score on the three subtests 3, 5 & 6 from TSVK, Siegmüller et al., 2010).

Panel A: Proportion of response accuracy is plotted on the y-axis.

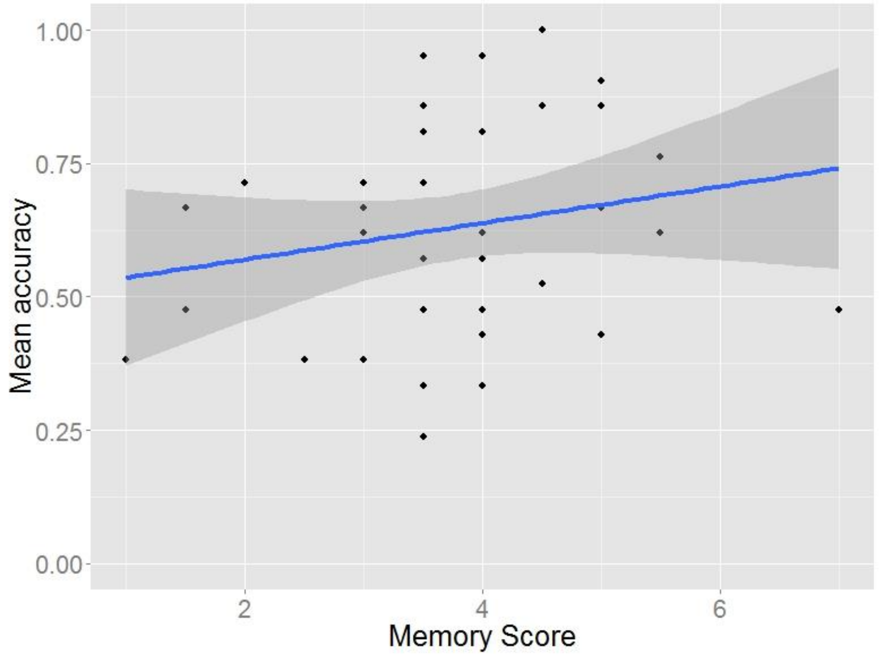

Panel B: Proportion of target looks is plotted on the y-axis.

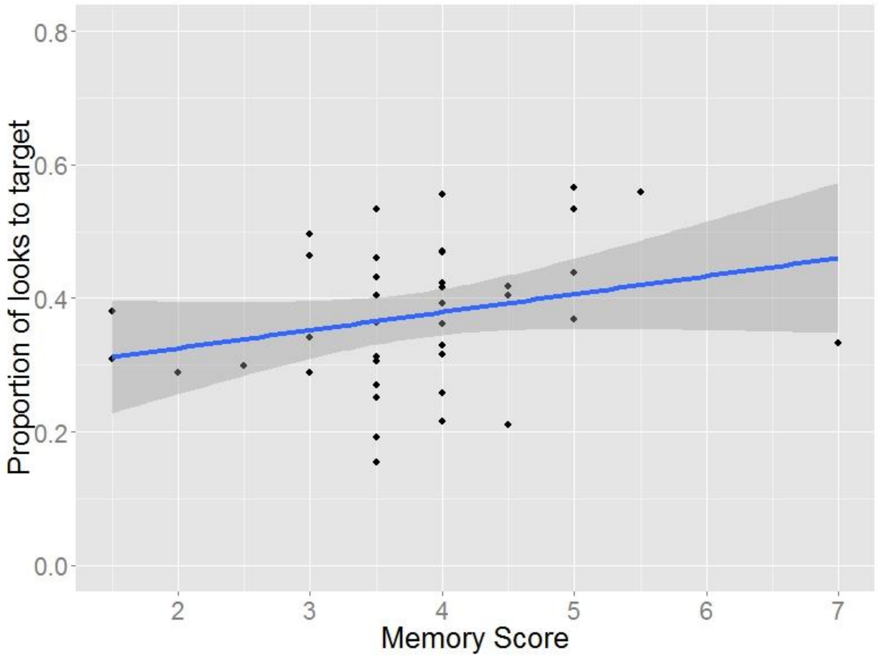

6. Model output – accuracy

Fixed effect	Coefficient	SE	z-value	p-value
Intercept	-.26	.34	-.75	.45
Condition OR+2DP vs. OR+3pro	-.12	.49	-.24	.81
Language Score	.36	.16	2.26	.02
Memory Score	.32	.23	1.39	.16
Language Score : Condition OR+2DP vs. OR+3pro	-.31	.13	-2.34	.02
Memory Score : Condition OR+2DP vs. OR+3pro	.13	.19	.67	.50
Language : Memory	-.18	.18	-1.04	.29
Language : Memory : Condition OR+2DP vs. OR+3pro	.10	.15	.67	.50

7. Model output – eye-tracking

Fixed effect	Coefficient	SE	t-value
Intercept	-.39	.05	-7.91
Time	17.51	.88	19.85
$Time^2$	-17.73	.89	-19.95
Condition OR+1pro vs. OR+2DP	-.82	.03	-30.88
Condition OR+2DP vs. OR+3pro	-.25	.03	-9.46
Memory Score	.09	.05	1.87
Language Score	.06	.03	1.98
Time : Condition OR+1pro vs. OR+2DP	-6.46	2.15	-2.99
$Time^2$: Condition OR+1pro vs. OR+2DP	31.89	2.18	14.65
Time : Condition OR+2DP vs. OR+3pro	-1.74	2.18	-.79
$Time^2$: Condition OR+2DP vs. OR+3pro	-.72	2.17	-.33
Time : Memory	-.85	.89	-.96
$Time^2$: Memory	-2.08	.88	-2.36
Memory : Condition OR+1pro vs. OR+2DP	-.13	.02	-5.07
Memory : Condition OR+2DP vs. OR+3pro	.04	.03	1.50
Time : Language	.14	.57	.25
$Time^2$: Language	-3.59	.58	-6.19
Language : Condition OR+1pro vs. OR+2DP	.08	.02	4.49
Language : Condition OR+2DP vs. OR+3pro	-.09	.02	-5.03

Memory : Language	.07	.04	1.73
Time : Memory: Condition OR+1pro vs. OR+2DP	-14.41	2.13	-6.75
$Time^2$: Memory: Condition OR+1pro vs. OR+2DP	-2.88	2.17	-1.33
Time : Memory: Condition OR+2DP vs. OR+3pro	8.86	2.19	4.04
$Time^2$: Memory: Condition OR+2DP vs. OR+3pro	3.37	2.11	1.59
Time : Language: Condition OR+1pro vs. OR+2DP	7.85	1.42	5.54
$Time^2$: Language: Condition OR+1pro vs. OR+2DP	-1.75	1.44	-1.21
Time : Language: Condition OR+2DP vs. OR+3pro	-4.56	1.43	-3.18
$Time^2$: Language: Condition OR+2DP vs. OR+3pro	5.41	1.45	3.74
Time : Memory: Language	.75	.74	1.02
$Time^2$: Memory: Language	-.44	.75	-.59
Memory: Language : Condition OR+1pro vs. OR+2DP	.05	.02	-2.19
Memory : Language: Condition OR+2DP vs. OR+3pro	.04	.02	1.70
Time : Memory: Language: Condition OR+1pro vs. OR+2DP	.72	1.77	.41
$Time^2$: Memory: Language: Condition OR+1pro vs. OR+2DP	3.88	1.82	2.13
Time : Memory: Language: Condition OR+2DP vs. OR+3pro	8.41	1.80	4.66
$Time^2$: Memory: Language: Condition OR+2DP vs. OR+3pro	-6.39	1.76	-3.63

8. Table of correlations between Memory Score, Language Score and overall performance in the experiment in terms of response accuracy

	Memory	Language	Mean Accuracy
Memory	1.0	.08	.17
Language		1.0	.28
Mean Accuracy			1.0

9. Table of correlations between Language Score, Memory Score and overall performance in the experiment in terms of proportion of target looks (PTL)

	Memory	Language	Mean PTL
Memory	1.0	-.005	-.19
Language		1.0	-.48
Mean PTL			1.0

10. Figure 3 – observed data

Proportion of target looks within the time window relevant for analysis, shown separately for each condition, divided by children's score on the memory tests (blue line = High Score; orange line = Low Score) and broken by their score on the language tests (top row = High Score; bottom row = Low Score). On the x-axis Time ranges from the offset of the relative pronoun until the end of the two-seconds long silence that followed the sentence. Two vertical dashed lines mark the critical chunks in the analysis window: (1) embedded subject DP (*ich* 'I'; *das Pferd* 'the horse'; *es* 'it'); (2) embedded verb (*jage/t* 'chase/s'); (3) post-sentential silence. The analysis of the eye-gaze data was performed on the entire time window shown in the plot (chunks 1-3).

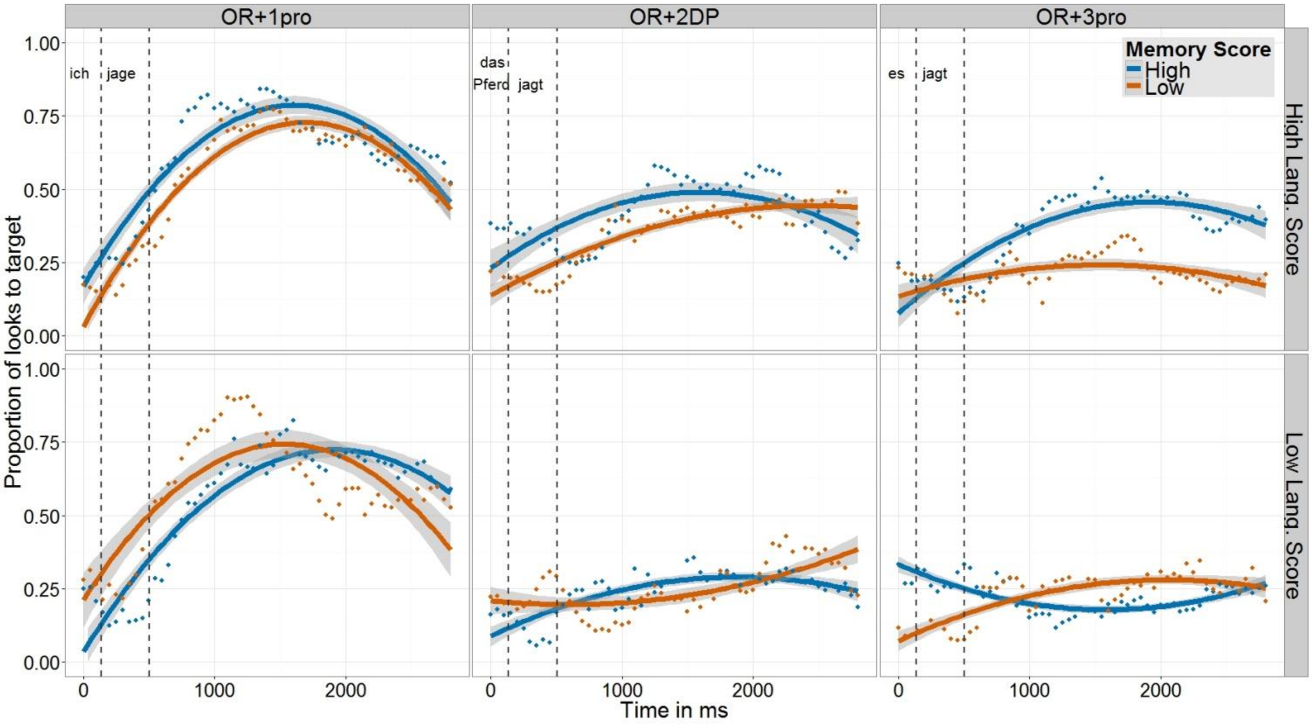

11. Figure 4 – observed data

Proportion of looks to the distractor figure within the time window relevant for analysis, shown separately for each condition, divided by children's score on the memory tests (blue line = High Score; orange line = Low Score) and broken by their score on the language tests (top row = High Score; bottom row = Low Score). On the x-axis Time ranges from the offset of the relative pronoun until the end of the two-seconds long silence that followed the sentence. Two vertical dashed lines mark the critical chunks in the analysis window: (1) embedded subject DP (*ich* 'I'; *das Pferd* 'the horse'; *es* 'it'); (2) embedded verb (*jage/t* 'chase/s'); (3) post-sentential silence. The analysis of the eye-gaze data was performed on the entire time window shown in the plot (chunks 1-3).

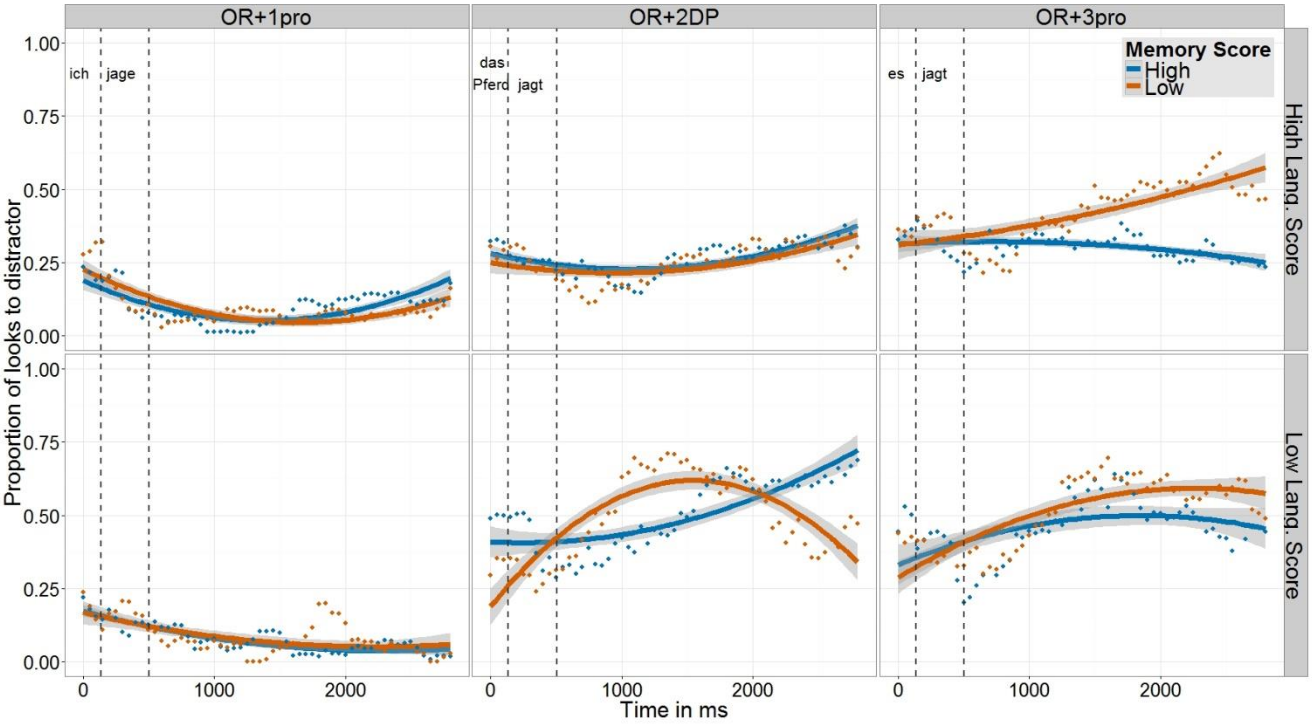

12. References

Siegmüller, Julia, Kauschke, Christina, van Minnen, Susanne and Bittner, Dagmar (2010). *Test zum Satzverstehen von Kindern: Eine profilorientierte Diagnostik der Syntax*. Munich, Germany: Urban & Fischer / Elsevier.
